# Time-adjusted direct medical costs of moderate-to-severe chronic graft-versus-host disease: a retrospective two-center cost analysis in Türkiye

**DOI:** 10.3389/fpubh.2026.1934364

**Published:** 2026-09-24

**Authors:** Ant Uzay, Yusuf Celik, Mesut Cimen, Muhammed Emre Guvey, Ecenur Aydemir, Ahmet Can Kucukkurt, Salim Yilmaz

**Affiliations:** 1 Department of Hematology, Acibadem Atakent Hospital, Istanbul, Türkiye; 2 Department of Medical Sciences, School of Medicine, Acibadem Mehmet Ali Aydinlar University, Istanbul, Türkiye; 3 Department of Healthcare Management, Faculty of Health Sciences, Acibadem Mehmet Ali Aydinlar University, Istanbul, Türkiye; 4 Department of Healthcare Management, Graduate School of Health Sciences, Acibadem Mehmet Ali Aydinlar University, Istanbul, Türkiye

**Keywords:** allogeneic hematopoietic cell transplantation, chronic graft-versus-host disease, cost determinants, direct medical costs, generalized linear model, time-adjusted cost analysis, Türkiye

## Abstract

**Introduction:**

Chronic graft-versus-host disease (cGVHD) imposes a substantial economic burden after allogeneic hematopoietic cell transplantation, yet evidence on cost determinants is limited, particularly from middle-income countries. The determinants of the daily intensity of direct medical costs in moderate-to-severe cGVHD were examined.

**Methods:**

Fifty patients treated between 2018 and 2023 at two private transplant centers in Türkiye were analysed retrospectively. Because treatment durations varied, cost was modeled as an intensity per day of observation using a Gamma generalized linear model with a log link and a log-time offset and robust (HC1) standard errors. Cost components and mortality were examined as exploratory analyses.

**Results:**

The mean total direct cost was 52,979 USD (SD 47,697), comprising medication (75.7%), inpatient (23.1%), and outpatient (1.3%) costs. Daily cost intensity was higher in male patients (cost ratio 2.163, 95% CI 1.451–3.224; +116% per day) and lower with older age (cost ratio 0.970 per year; −3.0% per year). Disease severity, transplant type, and organ involvement showed no association with daily cost intensity (joint Wald *p* > 0.5), indicating that severe disease raised cumulative cost mainly by extending treatment duration rather than the cost per day. Exploratory component-wise models suggested that medication intensity tracked disease characteristics whereas inpatient intensity tracked demographic factors. Mortality was 22% and underpowered.

**Discussion:**

Daily cost intensity was determined chiefly by male sex and younger age, whereas severity acted on cumulative cost through longer treatment. These hypothesis-generating findings support time-adjusted cost analysis in cGVHD.

## 1 Introduction

Allogeneic hematopoietic stem cell transplantation (allo-HCT) is a transformative treatment offering curative potential for patients with hematologic malignancies and immune-related disorders (1). However, one of the most frequent and burdensome complications following allo-HCT is chronic graft-versus-host disease (cGVHD), typically emerging after the first 100 days post-transplant (2).

cGVHD remains a prevalent and debilitating complication affecting 30–70% of patients undergoing allo-HCT globally, and cGVHD rates in Türkiye were reported between 35 and 45% depending on conditioning regimens, donor types, and prophylaxis strategies (3–5). International multicenter studies emphasize that cGVHD is not only a medical but also a systemic challenge, contributing to increased long-term morbidity, functional disability, and diminished quality of life through multisystem involvement, particularly of the skin, eyes, mouth, liver, and lungs (6, 7). Moderate to severe cGVHD is a major long-term complication of allo-HCT, leading to considerable non-relapse morbidity and mortality among transplant recipients (8, 9).

The economic implications of cGVHD are substantial across different healthcare systems. International studies reveal significant cost variations: Saito et al. (10) highlights that cGVHD substantially increases allo-HCT costs, with median expenses reaching 102,574 USD during the first 100 days and an additional 7,003 USD associated with cGVHD between 101 days and 1 year in Japan. Schain et al. (11) investigated cGVHD costs in Sweden, reporting cumulative three-year cohort-level expenses for patients with moderate-to-severe cGVHD at 47,811,835 EUR (approximately 51,636,781 USD), significantly higher than those without cGVHD. Yu et al. (12) reported median costs for steroid-resistant cGVHD patients amounting to 372,254 USD in the first year, which escalated to 532,673 USD by the second year in the United States. Maziarz et al. (13) conducted a study in the United States estimating the lifetime medical costs for allo-HCT patients at 942,373 USD to 1,247,917 USD, with cGVHD contributing 37–53% of the total expenses. Avenoso et al. (14) examined cGVHD costs in England, noting inpatient expenses of 17,339 GBP (approximately 22,153 USD) per patient-year compared to 8,548 GBP (approximately 10,926 USD) for those without cGVHD, with significantly higher costs for patients requiring high-cost therapies.

Although these studies provide an important evidence on the economic burden of cGVHD, most of the available cost data originate from high-income countries. Even though reimbursement structures and medicine prices are different, evidence from middle-income settings remains limited, and resource constraints may shape the economic burden of treatment differently across healthcare systems. This gap is particularly relevant for Türkiye, where prolonged immunosuppressive therapy, frequent hospital admissions, and comprehensive supportive care may generate substantial costs for the national insurance system and patients. Available evidence suggests that the financial burden of moderate-to-severe cGVHD in Türkiye follows the broader international pattern with costs substantially exceeding those observed in patients without cGVHD (10, 15–17).

Understanding the clinical outcomes and associated healthcare costs is crucial for enhancing care strategies and optimizing resource allocation. While cGVHD has been extensively studied in high-income countries, there is a lack of comprehensive data on its clinical and economic impact in Türkiye. The efforts to harmonize treatment protocols have led to the adoption of both first-line corticosteroid-based therapies and second-line options including extracorporeal photopheresis (ECP) and ruxolitinib (15, 18). Despite these interventions, outcomes remain variable, and this highlights the need for more standardized national treatment algorithms and improved early detection methods.

The growing complexity of allo-HCT recipient profiles, combined with regional disparities in transplant infrastructure, further underscores the need to integrate cGVHD-focused data into the background of research discussions. Studies detailing costs, healthcare resource utilization, and the progression of cGVHD in this specific context may improve the ability to develop effective healthcare policies and allocate resources efficiently. Understanding these dimensions is essential, given the growing prevalence of allo-HCT in Türkiye and the significant burden that cGVHD imposes on patients and the healthcare system. These considerations underscore the need to evaluate the prevalence and financial burden of cGVHD among allo-HCT patients in Türkiye, identify key cost drivers in treatment and resource utilization, and propose evidence-based strategies to optimize healthcare allocation and improve patient outcomes.

To the authors’ knowledge, this is the first study to quantify the patient-level direct medical cost of moderate-to-severe cGVHD and its determinants in Türkiye, and one of very few from any middle-income, single-payer setting; the existing cost-of-cGVHD evidence derives almost entirely from high-income health systems (14, 19, 20), whose pricing structures and reimbursement mechanisms differ substantially from those of Türkiye’s Social Security Institution.

## 2 Materials and methods

This study aims to evaluate the direct medical costs of moderate-to-severe cGVHD in Türkiye from a payer perspective by (1) estimating mean cost per patient and its distribution across medication, inpatient, and outpatient categories; (2) identifying clinical and demographic determinants of the daily intensity of cost, using a generalized linear model with a log-time offset that is appropriate for right-skewed cost data observed over unequal treatment durations; and (3) examining, as an exploratory analysis, whether these determinants operate differentially across cost components. The following research questions are addressed:

- What are the most frequently prescribed medicines and provided healthcare services in the studied hospitals for cGVHD patients?
- What is the mean cost per patient with cGVHD?
- What proportion of the overall cost for cGVHD patients is attributable to major categories such as medications, outpatient services, and hospitalizations?
- What are the clinical and demographic determinants of the daily intensity of cGVHD cost, and do these determinants differ across cost components?

These questions highlight the major cost categories, emphasize the significant share of medications and help identify underlying reasons such as treatment intensity or drug pricing, and also link clinical variables with costs to inform resource allocation and management strategies.

### 2.1 Study population and sample

This retrospective analytical study was conducted at two private transplant centers in Türkiye among a cohort of 437 adult patients who underwent bone marrow transplantation between January 1, 2018, and December 31, 2023, for various health conditions, primarily hematological malignancies, of whom 50 developed moderate-to-severe cGVHD and constituted the analytical sample. Both centers are private tertiary hospitals with dedicated adult allogeneic hematopoietic cell transplantation (allo-HCT) units located in Istanbul, Türkiye. The two centers differed substantially in transplant volume over the study period, contributing 385 and 52 of the 437 procedures, respectively.

Among the eligible population, 50 patients who developed cGVHD and had complete clinical and financial data were selected for detailed cost analysis. There were only four cGVHD cases in one of the two hospitals. The low number of cGVHD cases in this hospital is likely explained by its lower transplant volume (52 of 437), the absence of an SSI agreement during the study period, the predominance of international patients, the extensive use of alpha-beta depletion methods, and a preference for matched related donor (MRD) transplants. MRD transplants are generally associated with a lower risk of developing cGVHD. The selection of cGVHD patients was not random; rather, all patients with moderate-to-severe cGVHD and complete clinical and financial documentation during the study period were included, spanning the full range of disease severity and treatment duration. The sample is therefore a complete-case series of moderate-to-severe cGVHD at these two centers rather than a randomly drawn or statistically representative sample.

cGVHD severity was assessed by using the “Chronic GVHD Organ Scoring Form” protocol developed by the National Institutes of Health in 2014 (19) and adapted into Turkish by the Turkish Society of Hematology (20). This protocol allows patients to receive one of four possible scores [0-1-2-3], with higher scores indicating greater disease severity. Scoring data were extracted primarily from physicians’ clinical notes, where assessments had been documented to track disease progression and evaluate treatment response. To ensure scoring consistency, both participating hospitals applied the National Institutes of Health consensus criteria for diagnosing and grading cGVHD. However, it was observed that there were no standardized procedures such as inter-rater agreement protocols, formal scoring training, or harmonized data collection practices across hospitals. Although the Eastern Cooperative Oncology Group (ECOG) performance status was captured as part of the broader clinical assessment within the 2014 cGVHD evaluation process, ECOG-based performance scores were not systematically reported as a separate measure of patients’ functional status in routine clinical practice. Therefore, despite the recognized importance of standardized functional performance measures in characterizing patients’ overall clinical status, disease severity in the present study was classified according to the NIH cGVHD severity scores rather than ECOG performance grades. Accordingly, ECOG performance status was not included as an independent variable in the multivariable analyses.

### 2.2 Data and cost categories

This study followed the bottom-up gross-costing approach to estimate mean cost of cGVHD. Data collection involved three phases: identification, measurement, and valuation of resources. In the first step, aggregate resource items used by each patient were identified and classified into three major categories: medication, inpatient hospitalization, and outpatient services. In the second step, these resources were measured in natural units such as daily dose of prescribed medicine, number of hospitalizations, length of stay, or number of outpatient visits, etc. (21).

Prescriptions in PDF format were manually reviewed and converted into structured Excel spreadsheets for further analysis. The name and daily dose of medications prescribed for preventing cGVHD development, treating cGVHD, and addressing complications caused by cGVHD were categorized separately. Medications prescribed during outpatient visits were dispensed from community pharmacies rather than hospital pharmacies and were used by patients outside the hospital. All three cost components, namely medication, inpatient and intensive care, and outpatient visit costs, were valued exclusively using official Social Security Institution (SSI) reimbursement unit prices; no list prices, institutional charges, or out-of-pocket payments were used. To ensure that costs were expressed in constant prices and were not distorted by inflation or currency depreciation over the 2018–2023 study period, all resource utilization was valued using a single set of 2024 SSI reimbursement unit prices applied uniformly to every patient regardless of treatment year. Because all costs were therefore already expressed at the 2024 price level, no further inflation adjustment was required, and the resulting Turkish Lira amounts were converted to US Dollars using the 2024 average exchange rate (1 USD = 32.85 TRY) published by the Central Bank of the Republic of Türkiye (22) to facilitate international comparability. This study adopts a third-party payer perspective, specifically that of the Social Security Institution (SSI). Accordingly, the cost boundary comprises only direct medical costs reimbursed by the SSI: medication, inpatient and intensive care, daycare services, and outpatient services. Physician fees were not included because, in the private hospitals studied, they are billed separately and are not captured in the standardized invoices issued to the SSI. Indirect costs (such as productivity loss and caregiver burden) and out-of-pocket payments incurred at other facilities fall outside the payer perspective and were therefore not assessed by design rather than omitted inadvertently.

Hospitalization-related resources and their costs were derived from patient invoices issued to the SSI. These costs were categorized into four main areas: outpatient services; daycare services including intravenous immunoglobulin (IVIG), ECP and abatacept treatments; inpatient services; and intensive care unit (ICU) services. For each patient the observation window began at the onset of cGVHD and extended over the total treatment duration; this duration, in days, served as the denominator for the per-day cost analysis described in Section 2.3. Additional variables extracted for exploratory analysis included the primary diagnosis grouped into five categories (AML/MPAL, ALL/LBL, lymphoma, MDS/MPN, and CML) together with an acute myeloid leukemia indicator, the use of high-value targeted agents (ruxolitinib or ibrutinib), and all-cause mortality during follow-up.

### 2.3 Data analysis

Descriptive statistics were presented as mean ± standard deviation (SD) and median (interquartile range) for continuous variables and as frequencies and percentages for categorical variables. Group comparisons were performed using Mann–Whitney U and Kruskal–Wallis tests for continuous variables and chi-square or Fisher’s exact tests for categorical variables.

#### 2.3.1 Distributional assessment and GLM family selection

Healthcare cost data typically exhibit right-skewed distributions with variance proportional to the mean, violating ordinary least squares (OLS) assumptions. The distribution of total cGVHD costs was assessed for skewness and kurtosis. To empirically determine the appropriate GLM family, the Modified Park Test was applied, which regresses log-squared residuals on log-fitted values from an initial OLS model; the resulting coefficient (λ) indicates the variance function (λ ≈ 0: Gaussian; λ ≈ 1: Poisson; λ ≈ 2: Gamma; λ ≈ 3: inverse Gaussian) (23). The standard linear Park Test was extended with quadratic and cubic polynomial specifications to evaluate potential nonlinear variance–mean relationships. Final family selection was confirmed by comparing Akaike Information Criterion (AIC) and Bayesian Information Criterion (BIC) across competing GLM families (Gaussian, Gamma, and inverse Gaussian with alternative link functions).

#### 2.3.2 Primary model specification

A generalized linear model with a Gamma distribution and a log link was selected as the primary analytical framework. To account for the markedly different lengths of observation across patients, the natural logarithm of the total treatment duration in days was entered as a fixed offset, so that the modeled outcome is the expected cost per day of observation rather than the cumulative cost. Under this specification the exponentiated coefficients are interpretable as daily cost ratios (CR), representing the multiplicative change in the expected cost per day associated with a one-unit change in the predictor. The offset was preferred to entering treatment duration as a covariate, which would raise tautological concerns because longer treatment mechanically accumulates more resource consumption; expressing cost as an intensity per day of observation instead removes the dependence of cumulative cost on the length of follow-up. The model included gender, age at cGVHD diagnosis, disease severity (NIH 2014 consensus criteria [19]: score 1, score 2, score 3), transplant type (MRD, MUD, haploidentical), and the number of involved organ systems (1–3 vs. 4–7) as predictors. Multicollinearity was assessed using Generalized Variance Inflation Factors (GVIF), with adjusted values below 2.0 indicating acceptable levels.

#### 2.3.3 Handling of differential follow-up

Because the analytical cohort is a complete-case series assembled at two centers, patients were observed over widely differing treatment durations, and cumulative cost is mechanically higher in patients followed for longer. Modeling cumulative cost directly would therefore confound the clinical and demographic effects of interest with the length of observation, an issue analogous to immortal-time and differential-follow-up bias. Entering the logarithm of treatment duration as an offset reframes the outcome as the cost accrued per day of observation, which is comparable across patients regardless of how long each was followed. Treatment duration was consequently treated as the exposure window rather than as a covariate or an intermediate variable, and no mediation of clinical effects through treatment duration was estimated.

#### 2.3.4 Robust inference and model diagnostics

Heteroscedasticity-consistent standard errors (HC1) were computed using the sandwich estimator (24) and reported alongside naïve model-based standard errors. Given the small sample size, the analysis was repeated using HC4 standard errors (25), which apply a more aggressive leverage-based correction suited to small samples with influential observations; HC1 and HC4 yielded near-identical estimates with no change in inference for any predictor (Supplementary Table S6). A side-by-side comparison of naïve and robust estimates for all parameters is provided in Supplementary Table S8. Model diagnostics included deviance residual plots, Q–Q plots, and scale–location plots. Influential observations were identified using Cook’s distance with a threshold of 4/n.

#### 2.3.5 Component-wise exploratory models

To explore whether cost determinants operate differentially across expenditure categories, separate Gamma GLM models with the same log-time offset and predictor set as the primary model were fitted to the medication, inpatient, and outpatient cost components. Because disaggregation reduces the effective information available to each model and no adjustment for multiplicity was applied, these analyses are reported as exploratory and hypothesis-generating. The outpatient component model converged only with extended iterations and is likewise reported as exploratory.

#### 2.3.6 Sensitivity analysis

Model robustness was assessed by re-estimating all models after excluding influential observations identified via Cook’s distance. Cost ratio stability was evaluated using a threshold of <20% change between full-sample and trimmed-sample estimates.

#### 2.3.7 Mortality analysis

All-cause mortality was summarized descriptively and by NIH overall severity. Survival across severity groups was compared using Kaplan–Meier estimates with the log-rank test, and severity-adjusted associations were explored using Cox proportional-hazards and logistic models that included age. Because deaths were few and the score 1 group contained no events, these multivariable models were unstable owing to separation and are reported as exploratory only; a definitive survival analysis awaits exact death and last-contact dates.

All analyses were performed in R (version 4.5.2; R Core Team, 2025). Key packages included stats (GLM estimation), sandwich and lmtest (robust standard errors), car (GVIF), aod (joint Wald tests), survival (time-to-event analyses), moments (distributional assessment), and ggplot2 (visualizations). Statistical significance was set at α = 0.05.

## 3 Results

### 3.1 Patient characteristics and descriptive cost profile

The study identified a total of 437 Turkish citizens who underwent allo-HCT at the adult bone marrow transplant units of two private hospitals in Istanbul. Among these, 50 patients (11.4%) developed moderate-to-severe cGVHD and constituted the analytical sample. The mean age at cGVHD diagnosis was 44.1 years (SD 13.5), and 60% (30/50) were male. Matched related donor (MRD) transplants accounted for 40% of cases, with matched unrelated donor (MUD) and haploidentical transplants each accounting for 30%. Multiple organ involvement (≥2 organs) was observed in 92% of patients, and 52% had involvement of four or more organ systems. Table 1 summarizes the baseline characteristics of the cohort.

**Table 1:** Baseline patient characteristics (*N* = 50).

Characteristic	Value
Age, years, mean ± SD	44.1 ± 13.5
Male sex, *n* (%)	30 (60.0)
Primary diagnosis, *n* (%)	
AML/MPAL	18 (36.0)
ALL/LBL	16 (32.0)
Lymphoma	8 (16.0)
MDS/MPN	5 (10.0)
CML	3 (6.0)
Donor type, *n* (%)	
Matched related donor (MRD)	20 (40.0)
Matched unrelated donor (MUD)	15 (30.0)
Haploidentical	15 (30.0)
NIH overall severity score, *n* (%)	
Score 1	6 (12.0)
Score 2	13 (26.0)
Score 3	31 (62.0)
≥4 organ systems involved, *n* (%)	26 (52.0)
Targeted-agent use, *n* (%)	29 (58.0)
Follow-up, days, median [IQR]	724 [384–1,206]
Death, *n* (%)	11 (22.0)
Total direct cost, USD, mean ± SD	52,979 ± 47,697
Total direct cost, USD, median [IQR]	38,438 [21,942–64,509]

Among patients who developed cGVHD (*n* = 50), the mean duration of outpatient prophylaxis following transplantation was 113 days, ranging from 0 to 340 days. Notably, a duration of ‘0’ indicates that cGVHD symptoms manifested prior to hospital discharge, thereby precluding the initiation of outpatient prophylaxis. The average time from the transplantation to the onset of cGVHD was 132 days, ranging from a minimum of 19 to a maximum of 357. The total treatment duration, which defined the observation window used as the offset in the cost models, had a median of 724 days (IQR 384–1,206; range 66–2037).

Table 2 presents drug costs in two categories: prophylactic treatments administered to prevent cGVHD and therapeutic treatments for patients who developed cGVHD, further stratified by first recorded score, last recorded score, and clinical outcome. Given the chronic and variable nature of cGVHD, with fluctuations in severity over time, it was not feasible to classify patients according to fixed severity levels or standardized treatment durations; cost distributions were therefore presented according to more stable and classifiable characteristics. Mean prophylaxis costs were consistently lower than treatment costs across all patient groups, and the proportion of costs attributable to non-cGVHD drugs was considerably higher during the prophylaxis period. With the development of cGVHD, cGVHD-specific drugs became the dominant cost component within total medication expenditure.

**Table 2:** Medication costs and treatment duration by cGVHD score and outcome in outpatient patients (USD).

Scoring and clinical outcome	Cost of prophylaxis			Cost of treatment			Total duration of treatment
	cGVHD drugs	Other drugs	Total drugs	cGVHD drugs	Other drugs	Total drugs	
cGVHD score at time of diagnosis							
1 (n:11)	112.58	5607.58	**5720.16**	25403.18	20501.13	**45904.30**	790 days
2 (n:21)	87.14	4203.48	**4290.63**	20808.42	17851.51	**38659.93**	895 days
3 (n:15)	123.89	5357.39	**5481.28**	22190.57	23170.38	**45360.95**	783 days
Unknown (n:3)	94.53	359.12	**453.66**	343.32	568.85	**912.17**	550 days
Latest cGVHD scoring							
0 (n:14)	107.96	3704.42	**3812.38**	6287.73	18511.93	**24799.66**	1,036 days
1 (n:9)	101.80	5172.08	**5273.88**	15216.88	17208.65	**32425.53**	699 days
2 (n:10)	124.64	6552.46	**6677.10**	44656.57	28136.66	**72793.23**	995 days
3 (n:15)	98.81	4450.32	**4549.13**	25250.29	17204.00	**42454.29**	660 days
Unknown (n:2)	27.06	352.42	**379.48**	-	449.18	**449.18**	110 days
Clinical outcome							
Recovery (n:1)	45.88	5698.33	**5744.21**	252.44	20607.34	**20859.78**	1,100 days
Follow-up (n:34)	109.47	4799.87	**4909.34**	23824.07	19230.32	**43054.39**	920 days
Relapse (n:4)	58.98	901.40	**960.38**	5467.54	10479.28	**15946.83**	616 days
Death (n:11)	109.69	5354.11	**5463.80**	19832.65	21637.68	**41470.33**	548 days

USD: United States Dollar. Bold values indicate total drug costs, calculated as the sum of cGVHD drugs and other drugs within each subgroup.

When patients were grouped by their score at the time of diagnosis, treatment cost did not track the diagnosis-time score in a consistent way. Patients with a diagnosis score of 1 had the highest mean total treatment cost (45,904.30 USD) and prophylaxis cost (5,720.16 USD), while those with a score of 2 had the longest treatment duration (895 days). A similar lack of ordering was seen for the latest recorded score, where score 2 patients incurred the highest total treatment cost (72,793.23 USD) and prophylaxis cost (6,677.10 USD), and score 0 patients had the longest treatment duration (1,036 days), reflecting continued maintenance therapy. These single-timepoint scores capture severity only at one moment and do not reflect a patient’s overall disease course.

When stratified by clinical outcome, the single recovered patient had the longest treatment duration (1,100 days) and the highest prophylaxis cost (5,744.21 USD), whereas patients remaining under active follow-up carried the highest total treatment cost (43,054.39 USD; *n* = 34). Patients who died (*n* = 11) had shorter mean treatment durations (548 days) yet incurred comparatively high total treatment costs (41,470.33 USD). When patients were grouped by their overall NIH severity, total cost was higher in more severe disease, with the moderate (score 2, *n* = 13) and severe (score 3, *n* = 31) groups incurring broadly similar costs and the mild group (score 1, *n* = 6) substantially lower. As the time-adjusted analysis below shows, this gradient in cumulative cost reflected the longer treatment received in more severe disease rather than a higher cost incurred per day of care.

Figure 1 visualizes the distribution of the five most commonly used drugs in cGVHD treatment, categorized by organ involvement, multiple organ involvement, and clinical outcomes. Methylprednisolone, Tacrolimus, Prednisolone, Tacrolimus Monohydrate, and Ruxolitinib were the most frequently prescribed agents across all treatment phases. As shown in the heatmap, Methylprednisolone and Tacrolimus were the agents most commonly administered to patients with skin involvement (91.0 and 89.0%, respectively), with comparably high use in mouth involvement (94.0% for both). In patients with multi-organ involvement, prescribing patterns of these drugs remained consistent, and analysis by clinical outcome indicated that both Methylprednisolone and Tacrolimus were administered to most patients who remained under follow-up (88.0% each). These results highlight the consistent implementation of treatment protocols across different organ involvements and cGVHD severities.

**Figure 1:**
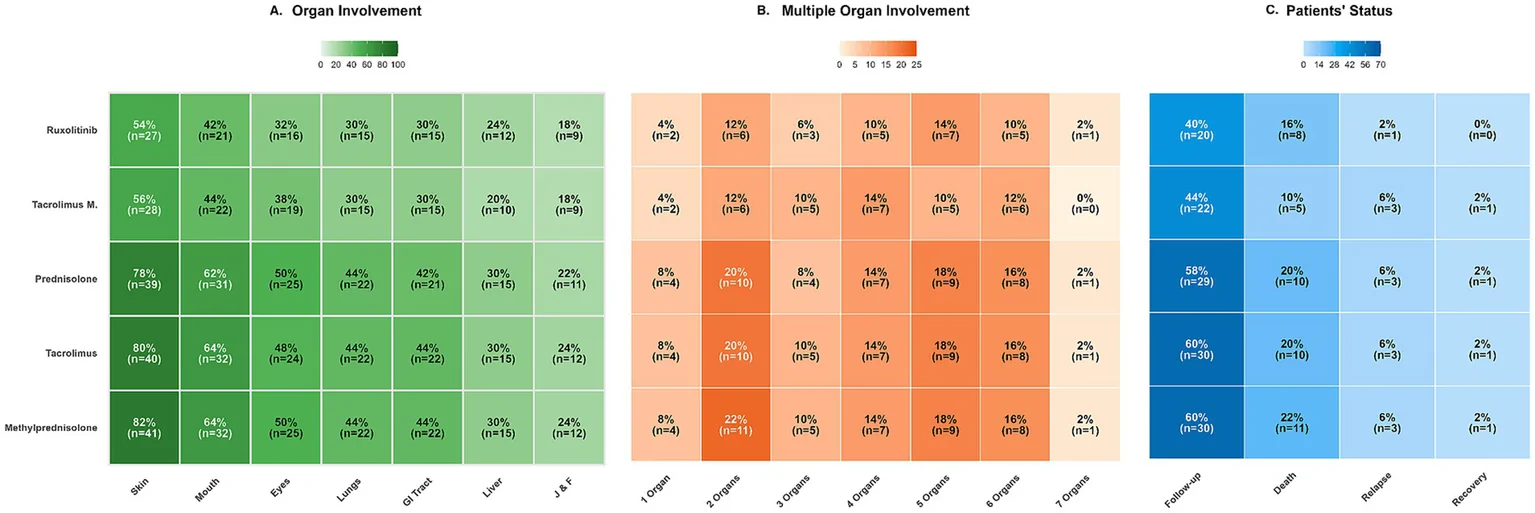
Most frequently used drugs in cGVHD treatment. **(A)** Frequency of involvement of each cGVHD organ system (skin, mouth, eyes, lungs, gastrointestinal tract, liver, and joints and fascia); **(B)** distribution of the number of simultaneously involved organ systems, from 1 to 7; **(C)** distribution of clinical outcome (under follow-up, death, relapse, and recovery); all shown by medication. Each cell reports the percentage of patients and the corresponding count (*n*); color intensity is proportional to the percentage, on a separate scale for each panel. Across all five agents, skin and mouth were the most frequently involved organ systems, multi-organ involvement (two or more organs) predominated, and the majority of patients remained under follow-up.

The most frequently prescribed cGVHD medications and their costs in preventing and treating cases were provided in Table 3. The average cost of outpatient prophylactic medications was calculated as 104.21 USD per patient. Among these, Tacrolimus (39.90 USD) and Ursodeoxycholic acid (38.52 USD) constituted the highest-cost drugs, while Methylprednisolone and Tacrolimus were the most commonly used. During the same period, the average cost of non-systemic agents addressing cGVHD-related complications was 4,627.89 USD, bringing the total drug cost to 4,732.10 USD.

**Table 3:** Costs of most frequently prescribed cGVHD medications in the outpatient setting (USD).

Medications	Costs			
	Mean	SD	Minimum	Maximum
Prophylaxis Medications in preventing cGVHD	104.21	78.96	0	329.95
Mycophenolate Mofetil	10.54	17.65	0	107.20
Methylprednisolone	10.51	11.73	0	43.84
Tacrolimus	39.90	47.63	0	159.25
Prednisolone	4.74	7.41	0	35.75
Ursodeoxycholic acid	38.52	67.24	0	249.25
Other Prophylaxis Medications	4627.89	5020.09	0	20114.66
Total cost of prophylaxis medications	**4732.10**	**5054.84**	**0**	**20319.21**
Medications for cGVHD treatment	21006.00	36799.22	0	163991.88
Other treatment medications	19087.40	16512.82	65.70	70556.52
Antimicrobial	10906.33	11357.75	31.07	55544.90
Skin	50.19	87.98	0	515.33
Mouth	3.24	7.01	0	31.90
Eyes	23.69	62.89	0	357.25
GI tract	505.92	1093.93	20.68	7695.40
Lungs	185.51	322.56	0	1750.31
Joints and facia	8.31	27.51	0	139.79
Other	7404.20	7904.19	3.46	32005.37
Total cost of treatment medications	**40093.40**	**46994.31**	**65.70**	**234548.41**

USD: United States Dollar; SD: Standard deviation. Bold values indicate the total medication costs for the prophylaxis and cGVHD treatment phases, respectively.

Drug costs increased significantly following the onset of cGVHD. While the mean cost of medications directly targeted at cGVHD management reached 21,006.00 USD, the average cost associated with other drugs administered during the treatment period was 19,087.40 USD. Among these drugs, antimicrobials accounted for the highest expenditure at 10,906.33 USD, followed by those categorized under the “other” group at 7,404.20 USD. Gastrointestinal agents (505.92 USD) and respiratory system medications (185.51 USD) also represented notable cost components. Although Ibrutinib was not among the most frequently prescribed drugs, it was associated with one of the highest individual drug costs. Although used in only 9 patients, Ibrutinib accounted for a disproportionate share of drug cost. The total average cost of all medications used during the outpatient cGVHD treatment reached 40,093.40 USD, approximately 8.5 times higher than the cost incurred during the prophylaxis phase.

Table 4 presents the categories of health service utilization and associated costs among hospitalized patients with cGVHD. The average number of hospitalizations per patient was 6.7, with a mean length of stay of 41.4 days. The mean number of hematology outpatient clinic visits was 51.88 per patient, corresponding to an average cost of 629.78 USD. The overall average cost of all outpatient clinic services amounted to 665.70 USD per patient.

**Table 4:** Utilization and costs of the most frequently used healthcare services by cGVHD patients (in USD).

Service/utilization measure	Costs		Utilization	
	Mean	Std. Dev.	Mean	Std. Dev.
Outpatient	**665.70**	**659.74**	**63.00**	**36.54**
Hematology	629.78	611.08	*51.88*	*28.03*
Other	35.92	72.57	*11.12*	*10.87*
Daycare	**6465.36**	**6996.80**	**29**	**20**
Intravenous immunoglobulin	4949.42	5821.71	*10.30*	*10.27*
Extracorporeal photopheresis	668.65	1666.23	*5.46*	*10.72*
Abatacept	22.84	132.56	*0.12*	*0.72*
Other	824.44	1959.35	*13.44*	*12.26*
ICU services	**138.38**	**339.06**	**0.50**	**0.76**
Other inpatient services	**3912.03**	**5371.81**	**–**	**–**
Total inpatient services	**12219.47**	**12615.01**	**–**	**–**
Number of hospitalizations	**–**	**–**	**6.70**	**6.08**
Length of stay	**–**	**–**	**41.40**	**35.32**

Values in bold denote the main service categories and their aggregate (subtotal or total) figures; values in italics denote the individual component items that make up each category. All costs are in USD. Std. Dev. = standard deviation; ICU = intensive care unit. A dash (–) indicates that the metric is not applicable to that row.

Among daycare services, IVIG administration accounted for the greatest financial burden. On average, patients underwent 10.3 sessions, resulting in a mean cost of 4,949.42 USD. This was followed by ECP, with an average usage of 5.46 sessions and an associated cost of 668.65 USD. The total cost of daycare services (6,465.36 USD) was significantly higher compared to other types of services. In contrast, other inpatient treatment services incurred an average cost of 3,912.03 USD while the mean cost of intensive care services was 138.38 USD. The total hospitalization cost per patient was found to be 12,219.47 USD. High standard deviation values across all service categories suggest substantial variability in both the intensity and cost of healthcare utilization, potentially reflecting differences in disease severity and duration of treatment.

Figure 2 summarizes the total costs associated with cGVHD across major expenditure categories. The average per-patient economic burden was calculated at 52,978.56 USD. Pharmacological treatments represented the largest share, accounting for 75.68% of the total cost (40,093.40 USD). Inpatient care comprised 23.06% (12,219.47 USD), whereas outpatient services constituted only 1.26% (665.70 USD) of the total expenditure.

**Figure 2:**
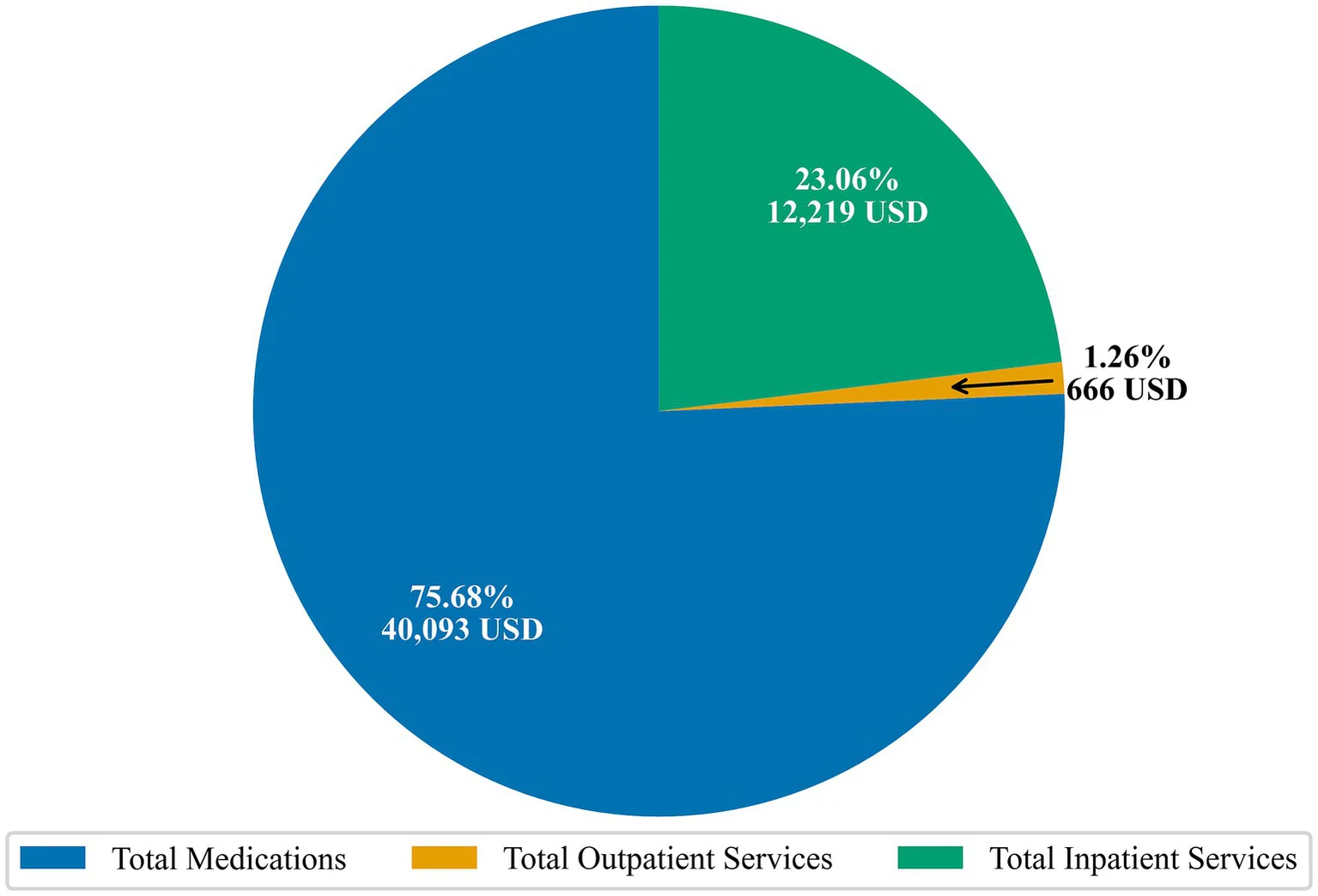
Summary of total cGVHD-related healthcare costs.

The analysis of cost distributions across categories demonstrated considerable variability, evidenced by a high standard deviation of 47,697.30 USD and a maximum expenditure of 244,429.05 USD. This variability highlights the substantial heterogeneity in treatment-related costs, likely driven by differences in cGVHD severity and duration among patients. Notably, drug-related expenditures alone exceeded 234,548.41 USD in certain cases. The total cost distribution exhibited marked right-skewness (skewness = 2.19, kurtosis = 8.15) with a long right tail extending beyond $150,000. These distributional characteristics indicate that the ordinary least squares assumptions of normally distributed, homoscedastic errors are violated, rendering standard linear regression inappropriate for this cost data. To identify a statistically appropriate framework that accounts for the mean–variance relationship inherent in healthcare expenditures, a systematic distributional assessment was conducted.

### 3.2 GLM family selection and model specification

The selection of an appropriate generalized linear model (GLM) family requires empirical assessment of how the variance of cost relates to its conditional mean, since different distributional families imply different variance functions. The Modified Park Test, which regresses the logarithm of the squared residuals on the logarithm of the fitted values, indicated that the variance increased with the mean in a manner consistent with the Gamma family rather than with the Gaussian or Poisson variance functions (Supplementary Table S3).

Comparison across candidate GLM configurations using both AIC and BIC supported this choice. The inverse-Gaussian specification achieved a slightly lower information criterion, but the Gamma family with a log link was retained because it yields directly interpretable cost ratios through exponentiated coefficients and guarantees positive support (Supplementary Table S4). Both criteria favored the Gamma and inverse-Gaussian families over the Gaussian alternative, and the agreement between AIC and the more conservative BIC indicates that family selection was not driven by the weaker complexity penalty of AIC alone. Visual assessment of the raw and log-transformed cost distributions further supported the Gamma selection (Supplementary Figure S1). Two additional specification checks were performed. First, multicollinearity among predictors was assessed using Generalized Variance Inflation Factors, with all adjusted values below 1.12, well within the conventional threshold of 2.0 (Supplementary Table S5). Second, naïve model-based standard errors were compared with heteroscedasticity-consistent (HC1) robust estimates; the two sets of standard errors were close in ratio and yielded identical significance classifications for every predictor, confirming that the Gamma variance function was well specified (Supplementary Table S6), with the complete naïve and robust inference presented in Supplementary Table S8. Residual diagnostic plots showed no systematic patterns (Supplementary Figure S2).

### 3.3 Determinants of the daily cost intensity of cGVHD

The primary analysis models the intensity of cost per day of observation, so that the effects of clinical and demographic characteristics are estimated free of the differences in follow-up length that would otherwise dominate a model of cumulative cost.

Table 5 presents the primary time-adjusted Gamma GLM. Exponentiated coefficients are interpreted as daily cost ratios (CR), representing the multiplicative change in the expected cost per day of observation associated with a one-unit change in the predictor. Robust HC1 standard errors are reported throughout, and the complete naïve and robust inference is provided in Supplementary Table S8.

**Table 5:** Determinants of the daily cost intensity of cGVHD (time-adjusted gamma GLM with log-time offset, *N* = 50).

Predictor	β (Robust SE)	Cost ratio	95% CI	*p*	Interpretation
Intercept	5.333 (0.347)	206.996	104.86–408.63	<0.001	—
Gender (Male vs. Female)	**0.771 (0.204)**	**2.163**	**1.451–3.224**	**<0.001**	**+116% per day**
Age (per year)	**−0.031 (0.007)**	**0.970**	**0.957–0.982**	**<0.001**	**−3.0% per year**
Severity: Score 1 vs. Score 3	0.044 (0.370)	1.045	0.506–2.157	0.906	Not significant
Severity: Score 2 vs. Score 3	−0.142 (0.213)	0.867	0.571–1.317	0.505	Not significant
Transplant: MUD vs. MRD	0.017 (0.241)	1.017	0.634–1.631	0.945	Not significant
Transplant: Haplo vs. MRD	−0.204 (0.240)	0.815	0.509–1.306	0.395	Not significant
Organs: 4–7 vs. 1–3	−0.033 (0.208)	0.967	0.643–1.454	0.873	Not significant

CR = daily cost ratio = exp(β). CI = 95% robust confidence interval (HC1). SE = robust standard error. Reference categories: Female, Score 3 cGVHD, MRD (matched related donor), 1–3 organs. Gamma GLM with a log link and a log-time offset, so the modeled outcome is cost per day of observation. AIC = 1,172.8; dispersion = 0.401. Joint Wald tests: disease severity χ^2^ = 0.49, df = 2, p = 0.78 (not significant); transplant type χ^2^ = 1.30, df = 2, p = 0.52 (not significant). Bold rows indicate predictors significant at α = 0.05.

In the time-adjusted model, the daily intensity of cost was determined chiefly by demographic characteristics. Male patients incurred 116% higher cost per day than female patients (CR = 2.163, 95% CI 1.451–3.224, *p* < 0.001), and each additional year of age at cGVHD diagnosis was associated with a 3.0% lower daily cost (CR = 0.970, 95% CI 0.957–0.982, *p* < 0.001). These were the only predictors that reached statistical significance.

Disease severity was not associated with the daily intensity of cost. Neither the score 1 versus score 3 contrast (CR = 1.045, *p* = 0.906) nor the score 2 versus score 3 contrast (CR = 0.867, *p* = 0.505) approached significance, and the joint Wald test for severity was clearly non-significant (χ^2^ = 0.49, df = 2, *p* = 0.78). This pattern indicates that the higher cumulative cost seen in more severe disease arose through a longer duration of treatment rather than through a higher cost incurred per day.

Transplant type (joint Wald χ^2^ = 1.30, df = 2, *p* = 0.52) and the involvement of four or more organ systems (CR = 0.967, *p* = 0.873) were likewise not associated with the daily intensity of cost. The absence of independent severity, transplant, and organ effects on the per-day scale localizes the measurable determinants of cost intensity to patient sex and age.

### 3.4 Component-wise exploratory analysis of cost components

The primary model identifies the determinants of overall daily cost intensity, but total cost aggregates three expenditure categories with potentially different economic mechanisms, namely medication (75.7% of total, mean 40,093 USD), inpatient hospitalization (23.1%, mean 12,219 USD), and outpatient visits (1.3%, mean 666 USD). To explore whether the identified determinants operate uniformly across components, separate time-adjusted Gamma GLM models with a log-time offset were fitted to the medication, inpatient, and outpatient components using the same predictor set as the primary model; the outpatient model converged only with extended iterations and is therefore reported as exploratory (Supplementary Table S15). Because these analyses disaggregate a small sample and were not adjusted for multiplicity, they should be interpreted with caution (Supplementary Tables S13–S15).

Medication intensity was associated primarily with disease and transplant characteristics rather than with sex. Relative to score 3, score 1 disease was associated with a 65.7% lower daily medication cost (CR = 0.343, *p* = 0.018). Compared with matched related donor transplantation, both matched unrelated donor (CR = 0.515, *p* = 0.014) and haploidentical (CR = 0.580, *p* = 0.032) transplantation were associated with a lower daily medication cost, and each additional year of age was associated with a 2.4% lower daily medication cost (CR = 0.976, *p* = 0.004). Male sex (CR = 1.464, *p* = 0.089) and involvement of four or more organ systems (CR = 1.394, *p* = 0.080) were not statistically significant. Pharmaceutical expenditure was concentrated in a small number of high-value targeted agents, chiefly ruxolitinib (used in 29 patients, 58%) and ibrutinib (9 patients, 18%), whose utilization and cost are detailed in Supplementary Table S12; a sensitivity analysis excluding these agents (Supplementary Table S16) preserved the principal determinants.

Inpatient intensity, by contrast, was associated primarily with demographic factors. Male sex was the dominant correlate, with male patients incurring roughly 3.4-fold higher daily inpatient cost than female patients (CR = 3.404, *p* < 0.001), and each additional year of age was associated with a 4.6% lower daily inpatient cost (CR = 0.954, *p* < 0.001). Matched unrelated donor transplantation was also associated with a higher daily inpatient cost relative to matched related donor transplantation (CR = 2.013, *p* = 0.037), whereas disease severity, haploidentical transplantation, and organ involvement were not significant. The divergence between the medication and inpatient components, with the former tracking disease and transplant characteristics and the latter tracking sex and age, should be interpreted cautiously given the exploratory nature of these models.

Figure 3 summarizes these component-level patterns. The lower daily medication cost associated with mild (score 1) disease is confined to the medication component (CR = 0.343), whereas the strong male and age associations are concentrated in the inpatient component. Subgroup-stratified cost composition is presented in Supplementary Figure S3.

**Figure 3:**
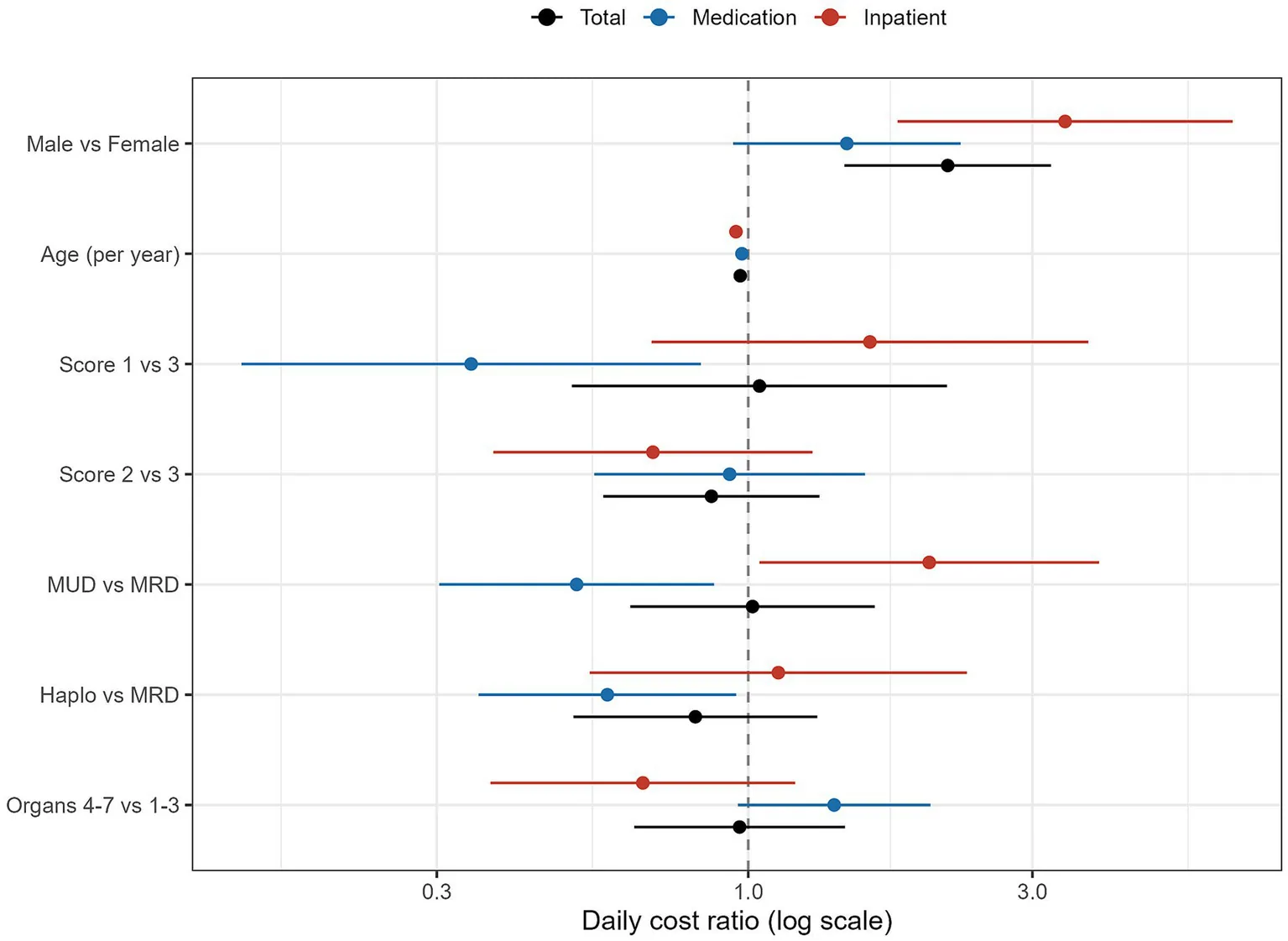
Daily cost ratios by cost component (exploratory). Gamma GLM with a log link, a log-time offset, and robust standard errors. The reference line at CR = 1.0 indicates no effect.

The strong male association with inpatient cost prompted examination of its mechanism. The higher inpatient cost in men was not attributable to more frequent or longer admissions; male patients had, if anything, fewer hospitalizations and a shorter total length of stay than female patients, and intensive care utilization was comparable between the sexes. The cost accrued per hospital day was instead higher in men, indicating that the elevated inpatient expenditure reflects greater per-admission intensity rather than a greater frequency or duration of hospitalization. These patterns are illustrated in Figure 4.

**Figure 4:**
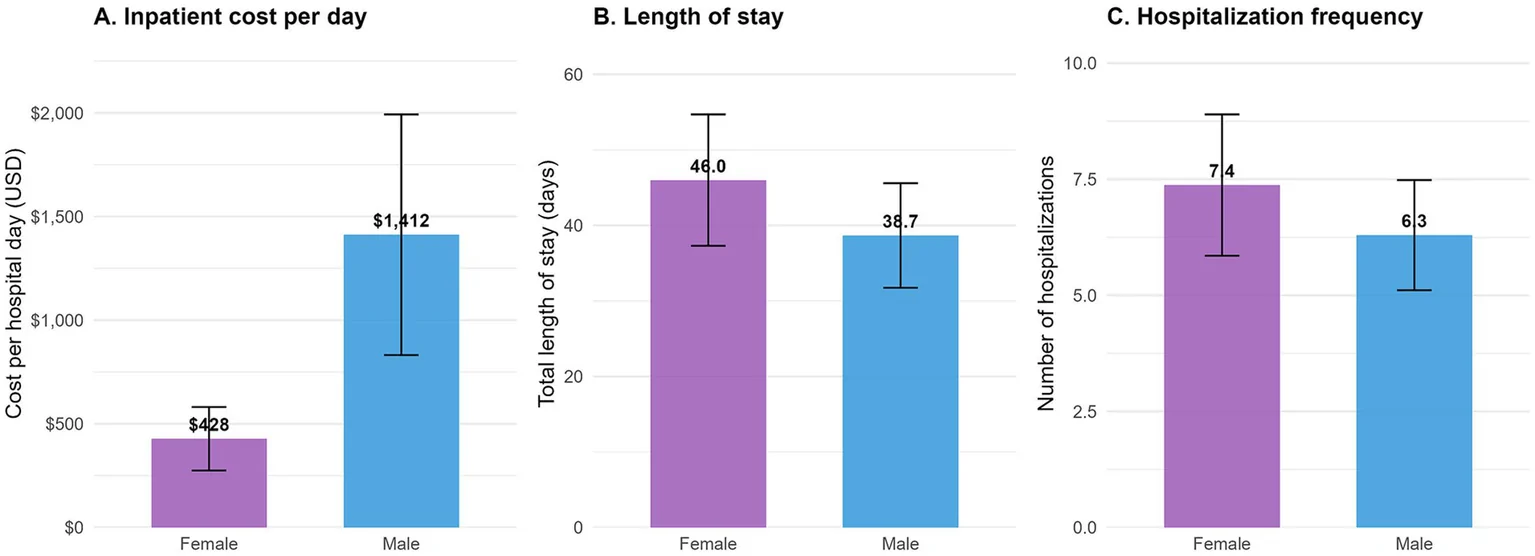
Mechanism of the gender difference in inpatient cost. **(A)** Mean inpatient cost per hospital day; **(B)** mean total length of stay; **(C)** mean number of hospitalizations, by gender. Bars show means; error bars show standard error. Male patients had shorter stays and fewer admissions but a markedly higher cost per hospital day, indicating that their higher inpatient cost reflects greater per-admission intensity rather than longer or more frequent hospitalization.

This per-diem dissociation localizes the source of the higher male cost to the intensity of care delivered during each admission rather than to admission frequency or duration, which suggests that cost-containment efforts targeting this differential should focus on the drivers of per-admission resource use, such as complication management and supportive care intensity, rather than on length-of-stay reduction (Figure 4).

### 3.5 Mortality (exploratory)

All-cause mortality during follow-up was 22% (11 of 50 patients). Deaths were distributed across the NIH overall severity groups as 0 of 6 in score 1, 2 of 13 in score 2, and 9 of 31 in score 3. Survival did not differ significantly across severity groups (Kaplan–Meier log-rank χ^2^ = 1.4, df = 2, *p* = 0.5). Because the score 1 group contained no deaths, severity-adjusted Cox proportional-hazards and logistic models were unstable owing to separation and are reported as exploratory only (Supplementary Table S22); in these models neither age nor severity was significantly associated with mortality. Given the small number of events these analyses are underpowered, and a definitive survival analysis awaits the collection of exact death and last-contact dates.

### 3.6 Sensitivity and robustness analyses

Given the relatively small sample (*N* = 50), individual observations can exert disproportionate influence on the estimates. Influential observations were identified using Cook’s distance with a pre-specified threshold of 4/*n* = 0.08. Five patients exceeded this threshold (Supplementary Table S17), each representing a clinically plausible but unusual cost–covariate combination (Supplementary Figure S4).

Re-estimation of the primary model after removing these observations (*n* = 45) left the significant determinants materially unchanged and preserved the direction of all estimates, confirming the stability of the male and age associations (Supplementary Table S18). A comprehensive summary of all hypothesis tests conducted in the study, including the distributional assessment, individual predictors, joint tests, component-wise models, and mortality analysis, is provided in Supplementary Table S20.

## 4 Discussion

The incidence of moderate to severe cGVHD among allo-HCT patients was found to be 11.4% in this study, and it falls into the reported broad range of 6 to 80% reported in the literature (26). Consistent with previous analyses (27), acute myeloid leukemia and acute lymphoblastic leukemia emerged as the most common underlying diagnoses among patients who developed cGVHD. Organ involvement demonstrated considerable clinical variability, with the skin (90.0%) being the most commonly affected site, followed by mouth (68.0%) and eyes (54.0%). The predominance of skin involvement is in line with findings by Martin et al. (28), who identified early inflammatory skin manifestations as a hallmark of cGVHD. Involvement of the GI tract (52.0%), lungs (48.0%), and liver (32.0%) reflects the systemic and multifocal nature of cGVHD progression as similarly emphasized by Farhadfar et al. (29) in their examination of clinical complications and associated healthcare burden. Less common involvement of organs such as joints and fascia (6.5%) underscores the need for increased clinical awareness of atypical presentations, which may otherwise be underdiagnosed or subjected to delayed intervention (30). Collectively, these findings underline the necessity for comprehensive organ-specific assessment and individualized therapeutic strategies to improve patient outcomes and mitigate complications in individuals affected by cGVHD.

Multiorgan involvement in cGVHD presents substantial clinical challenges, often necessitating comprehensive immunosuppressive regimens alongside targeted, organ-specific interventions. This complexity stems from its association with worse prognostic outcomes and higher mortality rates (27, 31). Analysis of treatment patterns highlights the predominant use of corticosteroids, particularly methylprednisolone and prednisolone, as the first-line therapies for inflammatory and immune-related complications across affected organs. Methylprednisolone was administered to the large majority of patients regardless of organ site, exceeding 90% across skin, mouth, eye, liver, and lung involvement. Among the organ sites affected in methylprednisolone-treated patients, skin (26.1%) and mouth (20.4%) involvement were the most frequent, followed by eye (15.9%), GI tract, and lung involvement (14.0% each). Tacrolimus, a calcineurin inhibitor, showed a very similar profile, with skin (25.8%) and mouth (20.6%) accounting for the largest shares, aligning with its established role in managing immune dysregulation in severe organ involvement (2, 30). Ruxolitinib, while not among the most commonly prescribed agents, was used in steroid-refractory cGVHD across a comparable distribution of organ sites, most often in patients with skin (25.5%) and mouth (19.8%) involvement. Its targeted use in these subgroups suggests that its therapeutic benefit becomes more pronounced in advanced or treatment-resistant disease manifestations (31). The co-administration of mycophenolate mofetil and sirolimus in select cases further reflects the clinical complexity of cGVHD management, indicating the need for multimodal immunosuppressive strategies to optimize therapeutic outcomes (32). Nonetheless, these combination therapies necessitate careful evaluation of both efficacy and toxicity profiles, as treatment response and adverse effects may vary substantially across different organ systems (33).

The present analysis quantifies the direct medical costs of cGVHD by categorizing expenditures according to disease severity scores recorded across consecutive hospital visits, thereby providing a detailed economic profile of the condition. Descriptively, cumulative cost per patient was lower in mild disease than in moderate or severe disease, with the moderate and severe groups broadly comparable. In the time-adjusted analysis, however, disease severity was not an independent determinant of the daily cost intensity (joint Wald *p* = 0.78), which indicates that the higher cumulative cost of more severe disease arose through a longer duration of treatment rather than a higher daily intensity of care. This modeling approach differs from several international cost analyses that relied primarily on descriptive comparisons or linear modeling approaches without explicitly accounting for the skewed and heteroscedastic nature of healthcare expenditure data (23, 34). In such settings, the independent contribution of severity may not be consistently demonstrated when distributional assumptions are not aligned with the empirical structure of cost data. These results suggest that distributionally appropriate specifications, in this case a Gamma family with a log link supported by the Modified Park Test, better capture the structure of skewed cost data than linear models, which are sensitive to residual heteroscedasticity and extreme observations.

The financial burden of cGVHD treatment was significant, with an average per-patient medication cost of 40,093.40 USD, 52% of which was attributed to cGVHD-specific medications. Notably, ruxolitinib and ibrutinib represented major cost components among the targeted agents, consistent with prior economic assessments and reflecting their established role in the treatment of steroid-refractory disease (27). The well-documented long-term adverse effects of corticosteroids, including increased susceptibility to infections and metabolic disturbances, further highlight the clinical need for effective alternative therapies (35). Beyond pharmacotherapy, cGVHD care imposes significant demands on healthcare resources, encompassing prolonged hospitalizations and intensive outpatient management. An average length of stay of 41 days, coupled with intensive care requirements and procedural interventions, underscores the resource-intensive nature of cGVHD (8).

Given that medications accounted for 75.7% of total direct medical costs in this cohort, pharmaceutical pricing and reimbursement policies are likely to remain important considerations in the economic burden of cGVHD. The increasing availability of generic medicines and, for biological therapies, biosimilars may influence treatment costs over time. Experience from European healthcare systems indicates that the economic impact of biosimilar entry depends not only on availability but also on national pricing, reimbursement, prescribing, and switching policies (36, 37). Future studies should therefore examine how changes in pharmaceutical markets and reimbursement policies affect the cost of cGVHD care in Türkiye.

These observations align with prior evidence demonstrating that GVHD-related complications and disease progression substantially increase healthcare utilization and costs (10, 12, 29, 38, 39). The wide variation in treatment duration across patients further suggests heterogeneity in clinical decision-making across providers and institutions, including differences in escalation protocols and authorization processes, consistent with claims-based analyses of cGVHD management (40). From a policy perspective, if treatment duration reflects provider-level practice variation rather than patient-level severity, it represents a potentially modifiable source of cost variability through pathway standardization and value-based care strategies (41).

The inverse relationship observed between patient age and the daily intensity of cost is consistent across both the primary model and the component-wise models, in which age was associated with both medication and inpatient cost intensity. This pattern may reflect the likelihood of less intensive therapeutic regimens or earlier mortality in older patients, resulting in shorter treatment courses and lower cumulative expenditures. This interpretation is reinforced by Weller et al. (42), who found that allo-HCT patients aged 60–79 had significantly poorer outcomes, potentially limiting the duration and intensity of cGVHD management. The consistency of the age effect across cost components suggests that it operates through a general reduction in treatment intensity rather than through a specific expenditure pathway.

Male sex was associated with a substantially higher daily cost intensity, incurring 116% higher cost per day, an association that remained robust to the removal of influential observations in the sensitivity analysis. The component-wise models indicated that this association arose almost entirely from the inpatient component rather than from medication expenditure. This dissociation suggests that the gender difference in total cost is not driven by differential prescribing patterns but by the intensity of inpatient resource use. Notably, this higher inpatient cost in men did not arise from more frequent or longer admissions; male patients had, if anything, fewer hospitalizations and a shorter total length of stay than female patients, and intensive care utilization was comparable between sexes. The cost accrued per hospital day was instead higher in men, indicating that the elevated inpatient expenditure reflects greater per-admission intensity, such as more resource-intensive supportive care, procedures, or management of acute complications, rather than longer or more frequent hospitalization. While the existing cGVHD literature has not consistently reported gender as a significant cost predictor, studies examining broader transplant populations have documented higher complication rates and healthcare utilization among male recipients (31, 43, 44). The specificity of this association to the inpatient component, apparent only when total cost was examined component by component, underscores the value of disaggregating total cost, as aggregate analyses may obscure the mechanisms through which demographic variables influence healthcare expenditure.

Beyond providing the first patient-level analysis of the direct medical costs of cGVHD and their determinants in Türkiye, and one of very few from any middle-income single-payer setting, this study makes two principal contributions. First, by modeling cost as an intensity per day of observation, it separates the determinants of how expensive care is per day from the determinants of how long care is provided, and shows that the daily intensity of cost is governed by patient sex and age rather than by disease severity, transplant type, or organ involvement; the higher cumulative cost of more severe disease is thus attributable primarily to a longer duration of treatment. Second, exploratory component-wise analyses suggest a divergence between expenditure categories, with medication intensity tracking disease and transplant characteristics and inpatient intensity tracking demographic factors. If confirmed, this divergence would carry practical implications, since interventions targeting medication cost, such as formulary optimization, biosimilar substitution, and severity-based treatment protocols, address disease characteristics, whereas interventions targeting hospitalization cost, such as early-discharge programs and outpatient management pathways, address demographic risk profiles. The concentration of pharmaceutical expenditure within a small number of high-value targeted agents further identifies a specific and actionable locus for cost containment in this resource-constrained setting.

These results emphasize the importance of incorporating real-time treatment duration and survival data into cost-monitoring frameworks, rather than relying solely on static indicators of disease severity at onset. This aligns with findings from a cost-utility analysis of ECP in cGVHD (45), which demonstrated that survival outcomes and changes in treatment duration were key determinants of total cost. Consistent with the present findings, the study showed that reducing prolonged immunosuppressant use and improving survival significantly influenced overall expenditure, highlighting the value of time-dependent and survival-adjusted metrics over static baseline severity measures (45). Accordingly, future cost-effectiveness assessments of cGVHD management should prioritize such metrics to more accurately capture the economic implications of treatment.

These findings point to directions for the Turkish single-payer system and comparable middle-income settings, all of which should be regarded as hypothesis-generating given the single-setting design. Because cumulative cost rises with severity largely through longer treatment, severe disease flags a higher total budget impact even though it does not raise the cost incurred per day, so the SSI could consider replacing uniform reimbursement with a two-band scheme tied to the NIH 2014 severity score at diagnosis, attaching a differentiated tariff or bundled payment to the moderate-to-severe band. Such a split is administratively simple and would make expenditure more predictable for payer and provider alike. The dominance of a handful of high-value agents, chiefly ruxolitinib and ibrutinib, means that prior-authorization pathways, biosimilar substitution, and formulary controls aimed specifically at these drugs would capture most of the potential savings without disturbing routine supportive care, an approach especially relevant where access to such agents is rationed by budget. The steep rise in cumulative cost with severity also makes upstream investment in GVHD prophylaxis at transplantation a credible cost-containment lever, since preventing progression to severe disease averts the most expensive downstream care. Because the component-wise analyses suggest that inpatient cost tracks demographic factors while medication cost tracks disease characteristics, containment strategies may need to be tailored to each component rather than applied as a single blanket measure.

## 5 Conclusion

This study identified the daily intensity of direct medical cost in moderate-to-severe cGVHD as being determined chiefly by male sex and younger age, whereas disease severity, transplant type, and organ involvement showed no independent association with the cost incurred per day. By expressing cost as an intensity per day of observation, the analysis distinguishes the determinants of daily cost from the length of treatment and indicates that the higher cumulative cost of more severe disease is generated largely by a longer treatment course rather than by a higher daily intensity of care. Exploratory component-wise analyses further suggested that medication intensity tracks disease and transplant characteristics while inpatient intensity tracks demographic factors, implying that cost containment may require component-specific rather than uniform strategies. Because cumulative cost rises steeply with severity, and because that rise operates mainly through treatment duration, severe disease flags a higher total budget impact that could be anticipated at diagnosis from the NIH 2014 global severity score. For Türkiye’s SSI framework this maps onto a possible two-band differentiated tariff or bundled-payment structure keyed to the mild versus moderate-to-severe distinction, which would be administratively simple and would improve cost predictability, while anticipated high-cost cases, in particular candidates for high-value targeted agents such as ruxolitinib, could be flagged at diagnosis for prospective budgeting. Strategies that reduce the incidence or severity of cGVHD, particularly optimized GVHD prophylaxis at transplantation, therefore represent a potentially powerful lever for containing the downstream economic burden. All of these implications are hypothesis-generating and require confirmation in larger, multicenter studies.

There are also limitations of this study. The most critical limitation is sample size and study setting. Fifty patients from two private centers in Istanbul restricts generalizability to public hospitals, other regions of Türkiye, and other healthcare systems, and the sample is weighted toward a single high-volume center. The retrospective design also limited the attribution of individual hospitalizations specifically to cGVHD, particularly for infections and other complications that may reflect cGVHD, its treatment, or concurrent clinical conditions. Hospitalization costs should therefore be interpreted as inpatient healthcare utilization occurring during the cGVHD treatment period rather than as costs with confirmed direct attribution to cGVHD for every admission. The retrospective design precluded control over data quality, and outpatient prescriptions were extracted from physician notes, which may be incompletely documented. Costs were calculated using 2024 SSI reimbursement rates applied uniformly across the 2018–2023 study period; while this expresses all costs at a constant 2024 price level and avoids distortion from inflation and currency depreciation, it does not capture year-to-year changes in drug pricing or reimbursement policy at the actual time of care. Score 1 cGVHD cases managed with non-cGVHD-specific medications may be underrepresented, as such cases are not always systematically documented. Physician fees were excluded because they are billed separately in private hospitals and not captured in standardized invoices, and the study captures only direct medical costs from the payer perspective, so indirect costs such as caregiver burden, productivity loss, and out-of-pocket payments elsewhere were not assessed. The offset used here is the total treatment duration rather than an exactly defined time at risk; this quantity will be refined once precise start, death, and last-contact dates are available, and the mortality analysis is correspondingly underpowered and affected by separation in the mild-disease group. Comorbidity burden and the hematopoietic cell transplantation comorbidity index were not available, and the dataset contains no center or hospital identifier, precluding formal adjustment for clustering. Accordingly, larger multicenter studies incorporating public-sector data are needed to validate the observed cost-structure patterns and to examine whether the determinant heterogeneity persists across institutional contexts.

## Data Availability

The datasets presented in this article are not readily available because the data presented in this study are available on request from the corresponding author due to restrictions related to third-party data ownership and usage permissions. Requests to access the datasets should be directed to yusuf.celik@acibadem.edu.tr.

## Glossary

allo-HCT: Allogeneic hematopoietic cell transplantation
AIC: Akaike information criterion
BIC: Bayesian information criterion
cGVHD: Chronic graft versus host disease
CI: Confidence interval
CR: Cost ratio
df: Degrees of freedom
ECP: Extracorporeal photopheresis
GLM: Generalized linear model
GVIF: Generalized variance inflation factor
HC1: Heteroscedasticity-consistent (Type 1) standard errors
Haplo: Haploidentical donor
IVIG: Intravenous immunoglobulin
MRD: Matched related donor
MUD: Matched unrelated donor
NIH: National Institutes of Health
OLS: Ordinary least squares
SD: Standard deviation
SE: Standard error
SSI: Social Security Institution
TRY: Turkish Lira
USD: United States Dollar
