# Supplementary material for: Time-adjusted direct medical costs of moderate-to-severe chronic graft-versus-host disease: a retrospective two-center cost analysis in Türkiye
Source: Front Public Health. 2026 Sep 24;14:1934364. doi: 10.3389/fpubh.2026.1934364 (PMC13645555; doi:10.3389/fpubh.2026.1934364)
Supplement: Supplementary file 1 [file Table_1.DOCX]

**Supplementary Materials**

***Time-Adjusted Direct Medical Costs of Moderate-to-Severe Chronic Graft-versus-Host Disease: A Retrospective Two-Center Cost Analysis in Türkiye***

This supplementary material provides detailed methodological documentation for the statistical analyses reported in the main manuscript. It is organized into nine sections covering variable definitions, distributional diagnostics and generalized linear model (GLM) family selection, model specification checks, full regression results for the primary model, cost component analysis, sensitivity analyses under influential observation removal, a comprehensive hypothesis testing summary, the statistical software environment used, and exploratory mortality and follow-up analyses. Each section includes the relevant tables and figures with full interpretive commentary. All reported p-values are two-sided, and statistical significance was evaluated at α = 0.05 unless otherwise noted.

**Contents and List of Sections and Supplementary Tables and Figures**

[Section 1: Abbreviations and Variable Definitions	3](#_Toc234533577)

[Table S1. Abbreviations Used in This Study.	3](#_Toc234533578)

[Table S2. Variable Definitions and Coding.	4](#_Toc234533579)

[Section 2: Distributional Assessment and Glm Family Selection	5](#_Toc234533580)

[Table S3. Modified Park Test Results For Variance Function Assessment.	5](#_Toc234533581)

[Table S4. Glm Family Comparison by İnformation Criteria.	5](#_Toc234533582)

[Figure S1. Cost Distribution and Stratified Boxplots.	6](#_Toc234533583)

[Section 3: Model Diagnostics and Specification Checks	7](#_Toc234533584)

[Table S5. Generalized Variance Inflation Factor Values for the Primary Model.	7](#_Toc234533585)

[Table S6. Naïve Versus Robust (HC1 and HC4) Standard Error Comparison For The Primary Time-Adjusted Model.	8](#_Toc234533586)

[Figure S2. Diagnostic Plots for the Gamma Glm (Log-Link) Total Cost Model.	9](#_Toc234533587)

[Section 4: Full Model Results	10](#_Toc234533588)

[Table S8. Primary Time-Adjusted Gamma Glm (Log-Time Offset) For The Daily Cost İntensity of CGVHD, Naïve and Robust İnference Comparison.	10](#_Toc234533589)

[Section 5: Cost Component Analysis	11](#_Toc234533590)

[Table S12. Utilization And Cost Of High-Value Targeted Agents (USD).	11](#_Toc234533591)

[Table S13. GLM Results For Medication Costs (75.7% of Total Cost).	12](#_Toc234533592)

[Table S14. GLM Results For İnpatient Hospitalization Costs (23.1% of Total Cost).	12](#_Toc234533593)

[Table S15. GLM Results For Outpatient Visit Costs (1.3% Of Total Cost).	13](#_Toc234533594)

[Figure S3. Cost Component Composition By Clinical And Demographic Subgroups.	13](#_Toc234533595)

[Table S16. Total-Cost Gamma Glm Sensitivity Analysis Excluding High-Value Targeted-Agent Expenditure.	14](#_Toc234533596)

[Section 6: Sensitivity Analyses	15](#_Toc234533597)

[Table S17. Clinical Characteristics of İnfluential Observations (Cook’s D > 4/N).	15](#_Toc234533598)

[Table S18. Cost Ratio Stability: Full Sample (N = 50) Versus Trimmed Sample (N = 45).	15](#_Toc234533599)

[Figure S4. Cook’s Distance Plot For the Total Cost Model.	16](#_Toc234533600)

[Table S19. Sensitivity Analysis: Full Versus Reduced (Parsimonious) Model Specification.	16](#_Toc234533601)

[Section 7: Hypothesis Testing Summary	18](#_Toc234533602)

[Table S20. Comprehensive Summary of all Hypothesis Tests.	18](#_Toc234533603)

[Section 8: Statistical Software And Reproducibility	19](#_Toc234533604)

[Table S21. R Packages Used For Statistical Analysis.	19](#_Toc234533605)

[Section 9: Mortality And Follow-Up Analyses	20](#_Toc234533606)

[Table S22. Mortality By CGVHD Severity.	20](#_Toc234533607)

[Figure S5. Kaplan–Meier Survival Curves by CGVHD Severity.	21](#_Toc234533608)

[Table S23. Follow-Up and Observation-Window Summary.	21](#_Toc234533609)

[References For Supplementary Material	22](#_Toc234533610)

Section 1: Abbreviations and Variable Definitions

This section provides a reference list of all abbreviations used throughout the supplementary material and the main manuscript, followed by the operational definitions and coding schemes for all variables included in the regression models. These definitions are essential for ensuring reproducibility and for interpreting the cost ratios reported in subsequent sections (Table S1).

Table S1. Abbreviations used in this study.

| **Abbreviation** | **Definition** |
| --- | --- |
| AIC | Akaike Information Criterion |
| allo-HCT | Allogeneic hematopoietic cell transplantation |
| BIC | Bayesian Information Criterion |
| cGVHD | Chronic graft-versus-host disease |
| CI | Confidence Interval |
| CR | Cost Ratio (exponentiated GLM coefficient) |
| ECP | Extracorporeal photopheresis |
| GI | Gastrointestinal |
| GLM | Generalized Linear Model |
| GVIF | Generalized Variance Inflation Factor |
| HC1 | Heteroscedasticity-consistent standard errors, type 1 |
| Haplo | Haploidentical donor |
| MRD | Matched related donor |
| MUD | Matched unrelated donor |
| NIH | National Institutes of Health |
| SD | Standard Deviation |
| SE | Standard Error |
| SSI | Social Security Institution |
| TRY | Turkish Lira |
| USD | United States Dollar |
| VIF | Variance Inflation Factor |

Table S2 presents the operational definitions, measurement types, and coding schemes for all variables entered into the regression models. The dependent variable is the total direct medical cost of cGVHD management, calculated as the sum of medication, inpatient, and outpatient cost components and converted from Turkish Lira to United States Dollars using annual average exchange rates. The independent variables include both demographic (gender, age) and clinical (disease severity, transplant donor type, organ involvement) characteristics as classified by the treating physicians according to NIH 2014 consensus criteria (Table S2).

Table S2. Variable definitions and coding.

| **Variable** | **Type** | **Coding** | **Description** |
| --- | --- | --- | --- |
| **Dependent Variable** |  |  |  |
| Total cGVHD cost | Continuous | USD | Sum of medication, inpatient, and outpatient costs; TRY converted to USD via annual average exchange rates (2018–2023) |
| **Demographic Variables** |  |  |  |
| Gender | Binary | 0 = Female, 1 = Male | Patient sex as recorded in medical records |
| Age at diagnosis | Continuous | Years | Age at time of cGVHD diagnosis |
| **Clinical Variables** |  |  |  |
| Disease severity | Ordinal | 1 = Mild, 2 = Moderate, 3 = Severe | NIH 2014 consensus criteria; Severe as reference |
| Transplant type | Nominal | MRD, MUD, Haplo | Donor type for allo-HCT; MRD as reference |
| Organ involvement | Binary | 1–3 organs, 4–7 organs | Number of organ systems affected; 1–3 as reference |
| **Treatment Variable** |  |  |  |
| Treatment duration | Continuous | Days | Total duration of cGVHD-directed pharmacotherapy |

NIH = National Institutes of Health; MRD = matched related donor; MUD = matched unrelated donor; Haplo = haploidentical donor; TRY = Turkish Lira; USD = United States Dollar. Disease severity was classified by the treating physician according to NIH 2014 consensus criteria incorporating both the number and severity of organ-specific manifestations. Organ involvement represents the total count of distinct organ systems (skin, mouth, eye, gastrointestinal, liver, lung, musculoskeletal) exhibiting cGVHD manifestations, dichotomized at the median.

Section 2: Distributional Assessment and GLM Family Selection

Healthcare cost data characteristically exhibit right-skewed distributions with heteroscedastic variance structures, rendering ordinary least squares regression inappropriate. To select the most suitable GLM family, the systematic framework proposed by Manning and Mullahy (2001) was followed, which combines empirical variance function assessment via the Modified Park Test with information-theoretic model comparison. This section documents the family selection process in full detail.

The Modified Park Test examines the relationship between the variance and mean of the dependent variable by regressing the squared raw residuals from an initial OLS model on the predicted mean. The resulting coefficient λ indicates the appropriate variance function: λ ≈ 0 suggests Gaussian (constant variance), λ ≈ 1 suggests Poisson (variance proportional to mean), λ ≈ 2 suggests Gamma (variance proportional to mean squared), and λ ≈ 3 suggests inverse Gaussian. To evaluate potential nonlinear variance–mean relationships, the standard linear specification was extended with quadratic and cubic polynomial terms, following Deb and Norton (2018). Table S3 presents the results.

Table S3. Modified Park Test results for variance function assessment.

| **Specification** | **λ (coefficient)** | **SE** | **p-value** | **Interpretation** |
| --- | --- | --- | --- | --- |
| Linear (Modified Park) | 3.50 | 0.475 | <0.001 | Variance rises steeply with the mean |

The Modified Park test regressed the log of the squared residuals on the log of the fitted mean. The estimated coefficient (λ = 3.50, p < 0.001) indicates that the variance rises steeply with the mean, well beyond the Gaussian (λ ≈ 0) and Poisson (λ ≈ 1) values and within the range spanned by the Gamma (λ ≈ 2) and inverse-Gaussian (λ ≈ 3) families. Because the test does not discriminate sharply between these two families at this sample size, the final family choice rested on information criteria and interpretability (Table S4).

To confirm the family selection empirically, the same predictor specification was estimated under five candidate GLM configurations and compared using the Akaike Information Criterion (AIC), Bayesian Information Criterion (BIC), and convergence status. Table S4 presents the results of this comparison.

Table S4. GLM family comparison by information criteria.

| **Model specification** | **AIC** | **BIC** | **Dispersion** | **Converged** |
| --- | --- | --- | --- | --- |
| Inverse Gaussian (log link) | 1159.4 | 1176.6 | 0.00 | Yes |
| Gamma (inverse link) | 1164.5 | 1181.7 | 0.385 | Yes |
| Gamma (log link) *[Selected]* | 1166.5 | 1183.7 | 0.387 | Yes |
| Gaussian (identity link) | 1217.9 | 1235.1 | — | Yes |
| Gaussian (log link) | — | — | — | No |

AIC = Akaike Information Criterion; BIC = Bayesian Information Criterion. The Gaussian model with log link failed to converge. Lower AIC and BIC values indicate better fit.

Both AIC and BIC ranked the inverse-Gaussian and Gamma families well above the Gaussian alternative. The inverse-Gaussian log-link model achieved the lowest AIC (1159.4) and BIC (1176.6), with the two Gamma specifications close behind (Gamma inverse link 1164.5/1181.7; Gamma log link 1166.5/1183.7); the Gaussian identity model was far worse (1217.9/1235.1) and the Gaussian log-link model failed to converge. The Gamma family with a log link was retained despite the marginally lower inverse-Gaussian criterion because its exponentiated coefficients are directly interpretable as cost ratios, it constrains predicted costs to be positive, and the inverse-Gaussian model offered no material improvement. The concordance of AIC and the more conservative BIC indicates the choice is not an artifact of the weaker AIC penalty.

To visually confirm the distributional characteristics of the cost data, a four-panel diagnostic figure was generated showing the raw cost distribution, the log-transformed distribution, and stratified boxplots by disease severity and gender.

Figure S1. Cost distribution and stratified boxplots.

The raw total cost distribution exhibited marked right-skewness (skewness = 2.21, kurtosis = 8.27) with a long right tail extending beyond $150,000, confirming that the Gaussian assumption of symmetric, constant-variance errors is untenable. Following log transformation, the distribution approximated normality, consistent with the Gamma family’s assumption that log-transformed means relate linearly to predictors. The severity-stratified boxplots revealed a clear monotonic cost gradient: severe cGVHD patients incurred substantially higher costs than moderate and mild patients, with considerable within-group heterogeneity in the severe category. The gender-stratified boxplots showed higher median and greater dispersion among males compared to females (Figure S1).

Section 3: Model Diagnostics and Specification Checks

Before interpreting the regression coefficients, it is essential to verify that the estimated model satisfies key specification assumptions, including absence of problematic multicollinearity and appropriate handling of potential heteroscedasticity. This section reports Generalized Variance Inflation Factor (GVIF) diagnostics and a comparison of naïve versus heteroscedasticity-consistent standard errors.

Multicollinearity was assessed using GVIF values, which generalize the standard VIF to accommodate categorical predictors with multiple degrees of freedom. The adjusted GVIF^1/(2·df)^ values were compared against the conventional threshold of 2.0, above which multicollinearity may distort coefficient estimates and inflate standard errors. Table S5 presents the GVIF results for the primary model (the preferred specification).

Table S5. Generalized Variance Inflation Factor values for the primary model.

| **Variable** | **GVIF** | **df** | **GVIF^1/(2·df)^** |
| --- | --- | --- | --- |
| Gender | 1.171 | 1 | 1.082 |
| Age | 1.100 | 1 | 1.049 |
| Disease severity | 1.341 | 2 | 1.076 |
| Transplant type | 1.311 | 2 | 1.070 |
| Organ involvement | 1.235 | 1 | 1.111 |

GVIF = Generalized Variance Inflation Factor; df = degrees of freedom for each predictor. The adjusted GVIF^1/(2·df)^ threshold of 2.0 is the conventional criterion for detecting problematic multicollinearity.

All adjusted GVIF^1/(2·df)^ values were well below 2.0, with the maximum being 1.111 for organ involvement. These results confirm that no problematic multicollinearity exists among the predictors, and coefficient estimates are not distorted by linear dependencies among the independent variables (Table S5).

Although the Gamma GLM with log link inherently accommodates mean–variance proportionality, residual heteroscedasticity may still exist if the assumed variance function is imperfectly specified. To guard against potential inference distortion, naïve (model-based) standard errors were compared with heteroscedasticity-consistent (HC1) sandwich estimators. If the Gamma variance function is correctly specified, naïve and robust standard errors should be similar; large discrepancies would indicate misspecification. Table S6 presents this comparison.

Table S6. Naïve versus robust (HC1 and HC4) standard error comparison for the primary time-adjusted model.

| Parameter | Naïve SE | HC1 SE | HC4 SE | p (HC1) | p (HC4) | Inference change |
| --- | --- | --- | --- | --- | --- | --- |
| Intercept | 0.350 | 0.347 | 0.360 | <0.001 | <0.001 | None |
| Male (vs Female) | 0.198 | 0.204 | 0.208 | <0.001 | <0.001 | None |
| Age (per year) | 0.007 | 0.007 | 0.007 | <0.001 | <0.001 | None |
| Mild (vs Severe) | 0.304 | 0.370 | 0.418 | 0.906 | 0.917 | None |
| Moderate (vs Severe) | 0.223 | 0.213 | 0.220 | 0.505 | 0.518 | None |
| MUD (vs MRD) | 0.227 | 0.241 | 0.256 | 0.945 | 0.948 | None |
| Haplo (vs MRD) | 0.235 | 0.240 | 0.246 | 0.395 | 0.406 | None |
| 4–7 organs (vs 1–3) | 0.199 | 0.208 | 0.217 | 0.873 | 0.878 | None |

HC1 and HC4 = heteroscedasticity-consistent standard errors (MacKinnon and White 1985; Cribari-Neto 2004). HC4 applies a more aggressive leverage-based adjustment recommended for small samples with influential observations. The HC4/HC1 ratio ranged from 1.006 to 1.062 across all parameters.

Naïve, HC1, and HC4 standard errors were closely similar across all parameters (HC4/HC1 ratio up to ~1.13 for the mild-severity contrast), and no predictor changed inference under any estimator. The agreement confirms that the Gamma variance function was well specified; HC1 is retained as the primary estimator.

Residual-based diagnostic plots were generated to visually assess model adequacy, including the randomness of deviance residuals, approximate normality, variance homogeneity, and the presence of influential observations.

Figure S2. Diagnostic plots for the Gamma GLM (log-link) total cost model.

The deviance residuals versus fitted values plot (Panel A) showed random scatter around zero with no discernible systematic pattern, suggesting adequate model specification. The normal Q–Q plot (Panel B) indicated approximate normality of deviance residuals with minor deviations in the upper tail attributable to the naturally heavy-tailed cost distribution. The scale–location plot (Panel C) showed relatively constant variance across the range of fitted values, confirming that the Gamma variance function adequately captures the mean–variance relationship. The Cook’s distance plot (Panel D) identified five observations (6, 12, 40, 47, and 48) exceeding the 4/n = 0.08 threshold, which are further examined in the sensitivity analysis (Section 6). Overall, the diagnostic plots support the adequacy of the Gamma GLM with log link for modeling cGVHD costs in this sample (Figure S2).

Section 4: Full Model Results

This section presents the complete regression output for the primary model specification. This model is a time-adjusted Gamma GLM with log link that includes a log-time offset, defined as the logarithm of each patient's observation window in days, so that the fitted quantity is the daily cost intensity of cGVHD management. All reported p-values use heteroscedasticity-consistent (HC1) robust standard errors. The exponentiated coefficients (exp(β)) are interpreted as cost ratios (CR), representing the multiplicative change in expected daily cost associated with a one-unit change in the predictor, holding other variables constant.

The primary model identifies which patient and disease characteristics predict the daily cost intensity of cGVHD independently of the length of the observation window. Adjusting for observation time through the log-time offset ensures that differences in follow-up duration across patients do not confound the estimated cost ratios. To facilitate complete methodological transparency, Table S8 presents the full results with both naïve (model-based) and HC1-robust standard errors and p-values side by side. The main text (Table 5) reports only the robust estimates; the dual presentation here allows readers to verify that inferential conclusions are consistent across both frameworks.

Table S8. Primary time-adjusted Gamma GLM (log-time offset) for the daily cost intensity of cGVHD, naïve and robust inference comparison.

| Predictor | β | Naïve SE | Robust SE | SE Ratio | CR | 95% CI LL | 95% CI UL | p (naïve) | p (robust) |
| --- | --- | --- | --- | --- | --- | --- | --- | --- | --- |
| Intercept | 5.333 | 0.350 | 0.347 | 0.991 | 206.996 | 104.856 | 408.630 | <0.001 | <0.001 |
| Male (vs Female) | 0.771 | 0.198 | 0.204 | 1.030 | 2.163 | 1.451 | 3.224 | <0.001 | <0.001 |
| Age (per year) | −0.031 | 0.007 | 0.007 | 0.940 | 0.970 | 0.957 | 0.982 | <0.001 | <0.001 |
| Mild (vs Severe) | 0.044 | 0.304 | 0.370 | 1.219 | 1.045 | 0.506 | 2.157 | 0.886 | 0.906 |
| Moderate (vs Severe) | −0.142 | 0.223 | 0.213 | 0.956 | 0.867 | 0.571 | 1.317 | 0.524 | 0.505 |
| MUD (vs MRD) | 0.017 | 0.227 | 0.241 | 1.063 | 1.017 | 0.634 | 1.631 | 0.942 | 0.945 |
| Haplo (vs MRD) | −0.204 | 0.235 | 0.240 | 1.021 | 0.815 | 0.509 | 1.306 | 0.385 | 0.395 |
| 4–7 organs (vs 1–3) | −0.033 | 0.199 | 0.208 | 1.043 | 0.967 | 0.643 | 1.454 | 0.867 | 0.873 |

CR = exp(β); 95% CI = Wald interval from robust (HC1) SE. Gamma GLM with log link and log-time offset; outcome = cost per day of observation. AIC = 1172.8; dispersion = 0.401; N = 50. Reference: Female, Severe (Score 3), MRD, 1–3 organs. SE Ratio = Robust/Naïve.

Two predictors reached significance under both naïve and robust inference. Male sex was associated with 116% higher daily cost (CR 2.163, 95% CI 1.451–3.224, p < 0.001) and each additional year of age with 3.0% lower daily cost (CR 0.970, 95% CI 0.957–0.982, p < 0.001). Disease severity, transplant type, and organ involvement showed no association with daily cost intensity; the severity contrasts (score 1 vs 3 CR 1.045, p = 0.906; score 2 vs 3 CR 0.867, p = 0.505) were far from significance. Naïve and robust standard errors were closely similar (ratios 0.94–1.22), confirming that the Gamma variance function was well specified.

Section 5: Cost Component Analysis

The total direct medical cost of cGVHD management comprises three components: medication costs (75.7% of total, mean $40,093 USD), inpatient hospitalization costs (23.1%, mean $12,219 USD), and outpatient visit costs (1.3%, mean $666 USD). To identify which patient and disease characteristics drive each component specifically, separate Gamma GLM models with log link and HC1 robust standard errors were estimated for each cost category using the same predictor set as the primary model. This component-wise analysis reveals that different predictors operate through fundamentally different cost pathways, a finding with direct implications for targeted cost-containment policy.

Medication costs represent the dominant cost component, constituting more than three-quarters of total cGVHD expenditure. This proportion is driven primarily by a small number of high-cost targeted agents. As shown in Table S12, ruxolitinib was administered to 29 patients (58.0%) and ibrutinib to 9 (18.0%); together these two agents alone accounted for 49.9% of all medication expenditure and 37.8% of total direct cost. Patients receiving ruxolitinib incurred 2.0-fold higher total costs than non-users (mean $67,371 versus $33,103).

Table S12. Utilization and cost of high-value targeted agents (USD).

| **Agent** | **Users, n (%)** | **Mean cost, all patients (USD)** | **Mean cost, users only (USD)** |
| --- | --- | --- | --- |
| Ruxolitinib | 29 (58.0) | 14,320 | 24,690 |
| Ibrutinib | 9 (18.0) | 5,683 | 31,570 |
| ECP | 15 (30.0) | 669 | 2,229 |
| IVIG | 46 (92.0) | 4,949 | 5,380 |
| Imatinib | 5 (10.0) | 284 | 2,839 |
| Sirolimus | 4 (8.0) | 186 | 2,320 |
| Etanercept | 2 (4.0) | 60 | 1,499 |
| Abatacept | 2 (4.0) | 28 | 712 |
| Everolimus | 1 (2.0) | 29 | 1,440 |

ECP = extracorporeal photopheresis; IVIG = intravenous immunoglobulin. Costs converted from Turkish Lira to United States Dollars at the 2024 average exchange rate (1 USD = 32.85 TRY). Mean cost across all patients reflects utilization frequency; mean cost among users reflects per-patient intensity.

The cost of these agents was highly concentrated. Ruxolitinib and ibrutinib were the principal drivers: although used in only a minority of patients, their high unit prices meant that together they consumed approximately half of all medication expenditure and over a third of total direct cost. The marked difference between the mean cost across all patients and the mean cost among users (for example, $5,683 versus $31,570 for ibrutinib) reflects the fact that these are second-line or salvage agents reserved for refractory disease rather than uniformly applied therapies. Extracorporeal photopheresis and intravenous immunoglobulin, although administered more frequently, contributed comparatively modestly to total expenditure on a per-patient basis. This concentration of cost in a small set of targeted agents indicates that medication expenditure in cGVHD is governed less by the breadth of supportive pharmacotherapy than by access to a few high-value therapies for severe or treatment-refractory disease (Table S12).

Table S13 presents the GLM results identifying which patient and disease characteristics drive medication costs.

Table S13. GLM results for medication costs (75.7% of total cost).

| Predictor | β | Robust SE | CR | 95% CI LL | 95% CI UL | p (robust) |
| --- | --- | --- | --- | --- | --- | --- |
| Male (vs Female) | 0.381 | 0.224 | 1.464 | 0.943 | 2.273 | 0.089 |
| Age (per year) | −0.024 | 0.008 | 0.976 | 0.960 | 0.992 | 0.004 |
| Mild (vs Severe) | −1.071 | 0.453 | 0.343 | 0.141 | 0.833 | 0.018 |
| Moderate (vs Severe) | −0.072 | 0.267 | 0.931 | 0.551 | 1.571 | 0.788 |
| MUD (vs MRD) | −0.663 | 0.271 | 0.515 | 0.303 | 0.876 | 0.014 |
| Haplo (vs MRD) | −0.545 | 0.254 | 0.580 | 0.353 | 0.955 | 0.032 |
| 4–7 organs (vs 1–3) | 0.332 | 0.190 | 1.394 | 0.961 | 2.023 | 0.080 |

Gamma GLM with log link and log-time offset; HC1 robust SE; N = 50.

Medication cost intensity was associated primarily with disease and transplant characteristics. Relative to severe disease, mild (score 1) disease carried 65.7% lower daily medication cost (CR 0.343, p = 0.018); matched unrelated (CR 0.515, p = 0.014) and haploidentical (CR 0.580, p = 0.032) transplantation carried lower daily medication cost than matched related donor; and each additional year of age was associated with 2.4% lower daily medication cost (CR 0.976, p = 0.004). Male sex (CR 1.464, p = 0.089) and involvement of four or more organ systems (CR 1.394, p = 0.080) did not reach significance.

Inpatient hospitalization accounts for approximately one-quarter of total cost. Unlike medication costs, hospitalization costs may reflect complications requiring admission, supportive care needs, and severity of acute episodes. Table S14 presents the GLM results for inpatient costs.

Table S14. GLM results for inpatient hospitalization costs (23.1% of total cost).

| Predictor | β | Robust SE | CR | 95% CI LL | 95% CI UL | p (robust) |
| --- | --- | --- | --- | --- | --- | --- |
| Male (vs Female) | 1.225 | 0.330 | 3.404 | 1.781 | 6.506 | <0.001 |
| Age (per year) | −0.047 | 0.010 | 0.954 | 0.935 | 0.973 | <0.001 |
| Mild (vs Severe) | 0.471 | 0.431 | 1.601 | 0.688 | 3.725 | 0.275 |
| Moderate (vs Severe) | −0.368 | 0.315 | 0.692 | 0.373 | 1.282 | 0.242 |
| MUD (vs MRD) | 0.700 | 0.335 | 2.013 | 1.044 | 3.882 | 0.037 |
| Haplo (vs MRD) | 0.116 | 0.372 | 1.123 | 0.542 | 2.329 | 0.754 |
| 4–7 organs (vs 1–3) | −0.407 | 0.301 | 0.665 | 0.369 | 1.199 | 0.175 |

Gamma GLM with log link and log-time offset; HC1 robust SE; N = 50.

Inpatient cost intensity was associated primarily with demographic factors. Male sex was the dominant correlate (CR 3.404, 95% CI 1.781–6.506, p < 0.001) and each additional year of age was associated with 4.6% lower daily inpatient cost (CR 0.954, p < 0.001). Matched unrelated donor transplantation was associated with higher daily inpatient cost than matched related donor (CR 2.013, p = 0.037), whereas disease severity, haploidentical transplantation, and organ involvement were not significant.

Outpatient costs, although representing only 1.3% of total expenditure, provide insight into ambulatory care patterns. One patient had zero outpatient costs (adjusted by adding $1 for Gamma GLM compatibility). Table S15 presents the GLM results.

Table S15. GLM results for outpatient visit costs (1.3% of total cost).

| Predictor | β | Robust SE | CR | 95% CI LL | 95% CI UL | p (robust) |
| --- | --- | --- | --- | --- | --- | --- |
| Male (vs Female) | 0.954 | 0.294 | 2.595 | 1.460 | 4.613 | 0.001 |
| Age (per year) | −0.039 | 0.012 | 0.962 | 0.940 | 0.983 | <0.001 |
| Mild (vs Severe) | 1.199 | 0.479 | 3.318 | 1.299 | 8.476 | 0.012 |
| Moderate (vs Severe) | 0.041 | 0.490 | 1.042 | 0.399 | 2.724 | 0.933 |
| MUD (vs MRD) | 0.621 | 0.383 | 1.860 | 0.878 | 3.944 | 0.105 |
| Haplo (vs MRD) | 0.490 | 0.442 | 1.633 | 0.687 | 3.884 | 0.267 |
| 4–7 organs (vs 1–3) | −0.222 | 0.296 | 0.801 | 0.448 | 1.432 | 0.454 |

Gamma GLM with log link and log-time offset; HC1 robust SE; N = 50. The model converged only with extended iterations; estimates are exploratory.

The outpatient component (1.3% of total cost) required extended iterations to converge and is reported only as exploratory. Higher daily outpatient cost was associated with male sex (CR 2.595, p = 0.001) and with mild disease (CR 3.318, p = 0.012), and lower cost with older age (CR 0.962, p < 0.001); the positive mild-disease association is consistent with milder patients being managed predominantly in the ambulatory setting. Given the small absolute size of this component and the fragile convergence, these estimates should be interpreted with caution.

To visualize the cost composition patterns across clinically defined subgroups, a stacked bar chart was generated showing the absolute and proportional contribution of each cost component stratified by disease severity, gender, and organ involvement category.

Figure S3. Cost component composition by clinical and demographic subgroups.

The stacked bar chart confirms the quantitative findings from the component GLMs. Medication costs dominate across all subgroups, but the relative proportions shift meaningfully: severe cGVHD patients show a higher medication share, while the inpatient proportion is relatively constant across severity levels. Males show higher absolute costs in all three components, with the inpatient differential being particularly pronounced. Patients with 4–7 organ involvement exhibit substantially higher medication costs compared to those with 1–3 organs, while their inpatient costs are similar, consistent with the regression findings that organ complexity primarily drives pharmaceutical expenditure rather than hospitalization (Figure S3).

Because medication expenditure was so heavily concentrated in a few high-value agents, an additional sensitivity analysis was performed to determine whether the determinant structure of total cost was merely an artifact of this concentrated spending. The primary total-cost model was re-estimated after subtracting all expenditure on high-value targeted agents (ruxolitinib, ibrutinib, sirolimus, everolimus, imatinib, etanercept, and abatacept), reducing mean total cost from $52,979 to $32,389. The results are presented in Table S16.

Table S16. Total-cost Gamma GLM sensitivity analysis excluding high-value targeted-agent expenditure.

| Predictor | β | Robust SE | CR | 95% CI LL | 95% CI UL | p (robust) |
| --- | --- | --- | --- | --- | --- | --- |
| Male (vs Female) | 0.834 | 0.262 | 2.302 | 1.378 | 3.846 | 0.002 |
| Age (per year) | −0.036 | 0.007 | 0.965 | 0.951 | 0.979 | <0.001 |
| Mild (vs Severe) | 0.262 | 0.346 | 1.299 | 0.659 | 2.562 | 0.450 |
| Moderate (vs Severe) | −0.219 | 0.206 | 0.804 | 0.537 | 1.204 | 0.289 |
| MUD (vs MRD) | 0.294 | 0.272 | 1.341 | 0.788 | 2.284 | 0.280 |
| Haplo (vs MRD) | 0.164 | 0.267 | 1.179 | 0.699 | 1.988 | 0.538 |
| 4–7 organs (vs 1–3) | −0.307 | 0.259 | 0.736 | 0.443 | 1.221 | 0.235 |

Outcome = total direct cost minus high-value targeted-agent expenditure, per day of observation (mean total cost reduced from $52,979 to $32,389). Gamma GLM with log link and log-time offset; HC1 robust SE; N = 50.

After excluding all expenditure on high-value targeted agents (mean total cost reduced from $52,979 to $32,389), the daily-intensity determinants were unchanged in structure. Male sex (CR 2.302, p = 0.002) and younger age (CR 0.965, p < 0.001) remained the only significant predictors, and disease severity remained non-significant (score 1 vs 3 CR 1.299, p = 0.450), consistent with the primary model. This confirms that the sex and age associations are not artifacts of concentrated high-value drug spending.

Section 6: Sensitivity Analyses

With a sample of 50 patients, individual observations can exert disproportionate influence on regression estimates. To assess the robustness of the findings, influential observations were identified using Cook’s distance with the conventional threshold of 4/n (= 0.08 for n = 50), characterized clinically, and the primary model was re-estimated after their removal. The criterion for stability was defined a priori as less than 20% change in cost ratio magnitude, with no qualitative reversal of statistical significance.

Five observations exceeded the Cook’s distance threshold in the total cost model (observations 6, 12, 40, 47, and 48). Table S17 characterizes these patients.

Table S17. Clinical characteristics of influential observations (Cook’s D > 4/n).

| **Obs.** | **Cook's D** | **Cost (USD)** | **Sex** | **Age** | **Severity** | **Organs** | **Follow-up (days)** | **Reason for influence** |
| --- | --- | --- | --- | --- | --- | --- | --- | --- |
| 6 | 0.080 | 46,634 | Female | 21 | Severe | 1–3 | 260 | High cost over short follow-up (high daily cost) |
| 12 | 0.118 | 143,514 | Male | 62 | Moderate | 4–7 | 1,208 | Very high total cost |
| 40 | 0.114 | 16,182 | Male | 61 | Mild | 4–7 | 994 | Low cost despite extensive organ involvement |
| 47 | 0.097 | 13,270 | Female | 45 | Mild | 1–3 | 1,431 | Low daily cost over long follow-up |
| 48 | 0.250 | 22,153 | Male | 41 | Mild | 1–3 | 66 | Very short follow-up, high daily cost |

Cook’s D threshold = 4/n = 4/50 = 0.08. All five observations represent clinically plausible but unusual cost–covariate combinations that exert leverage on regression coefficients due to their discordance between predicted and observed costs.

Five observations exceeded the Cook's distance threshold (4/n = 0.08) in the time-adjusted model. Each represents a clinically plausible but unusual combination of cost, follow-up length, and covariates, most reflecting extreme daily-cost values arising from very short or very long observation windows. None warrant exclusion on clinical grounds; their removal is used only to test robustness.

Table S18. Cost ratio stability: full sample (n = 50) versus trimmed sample (n = 45).

| **Predictor** | **Full CR** | **Full p** | **Trim CR (n=45)** | **Trim p** | **CR change (%)** | **Stable (<20%)?** |
| --- | --- | --- | --- | --- | --- | --- |
| Male (vs Female) | 2.163 | <0.001 | 1.985 | 0.001 | −8.2% | Yes |
| Age (per year) | 0.970 | <0.001 | 0.973 | <0.001 | +0.4% | Yes |
| Mild (vs Severe) | 1.045 | 0.906 | 1.193 | 0.510 | +14.2% | Yes |
| Moderate (vs Severe) | 0.867 | 0.504 | 0.800 | 0.230 | −7.8% | Yes |
| MUD (vs MRD) | 1.017 | 0.945 | 0.950 | 0.809 | −6.5% | Yes |
| Haplo (vs MRD) | 0.815 | 0.395 | 0.799 | 0.347 | −2.0% | Yes |
| 4–7 organs (vs 1–3) | 0.967 | 0.873 | 1.091 | 0.681 | +12.8% | Yes |

CR = cost ratio from Gamma GLM with log link and HC1 robust SE. Stability criterion: < 20% change in CR magnitude.

All seven predictors met the pre-specified stability criterion (<20% change in CR). The two significant determinants, male sex and age, retained their direction, magnitude, and significance after removing the five influential observations (male CR 2.163 → 1.985, p = 0.001; age CR 0.970 → 0.973, p < 0.001), and no non-significant predictor became significant. The time-adjusted findings are therefore robust to influential observations.

Cook’s distance was plotted for each observation to visualize the distribution of influence across the sample and to identify the specific observations exceeding the diagnostic threshold.

Figure S4. Cook’s distance plot for the total cost model.

The Cook’s distance plot reveals that the five flagged observations (6, 12, 40, 47, and 48) are clearly separated from the remaining 45 observations, all of which fall well below the 4/n threshold. The majority of observations cluster near zero influence, indicating that the regression estimates are primarily determined by the general pattern in the data rather than by individual extreme cases (Figure S4).

A further sensitivity analysis addressed the ratio of observations to estimated parameters. The primary model estimates 8 parameters from 50 observations, corresponding to approximately 6.3 observations per parameter, below the conventional 10–20 guideline for generalized linear models. To assess whether the principal findings were stable under a more parsimonious specification, the model was re-estimated retaining only the demographic and severity predictors (gender, age, and disease severity) and removing transplant type and organ involvement, which were not statistically significant in the primary model. This reduced model estimates 5 parameters, yielding exactly 10 observations per parameter. Table S19 compares the shared coefficients between the full and reduced specifications.

Table S19. Sensitivity analysis: full versus reduced (parsimonious) model specification.

| **Model** | **Predictors** | **AIC** | **BIC** |
| --- | --- | --- | --- |
| Full | sex, age, severity, transplant, organ | 1172.8 | 1190.0 |
| Reduced | sex, age, severity | 1167.8 | 1179.3 |

Reduced-model cost ratios: Male CR 2.150 (p < 0.001), Age CR 0.971 (p < 0.001), Mild (S1 vs S3) CR 1.120 (p = 0.754), Moderate (S2 vs S3) CR 0.809 (p = 0.265).

A parsimonious specification retaining only sex, age, and severity achieved a lower AIC (1167.8 vs 1172.8) and BIC (1179.3 vs 1190.0) than the full model. Sex (CR 2.150, p < 0.001) and age (CR 0.971, p < 0.001) remained significant and severity remained non-significant (score 1 vs 3 CR 1.120, p = 0.754), confirming that the significant determinants are stable to model simplification.

Section 7: Hypothesis Testing Summary

Table S20 provides a comprehensive summary of all formal statistical tests conducted in this study, organized into three groups: distributional assessment and model selection, individual predictor hypothesis tests, and diagnostic and robustness checks. This consolidated presentation enables readers to evaluate the complete evidentiary basis for the reported conclusions. All individual predictor tests used robust (HC1) standard errors, joint tests for multi-level categorical variables used Wald χ² statistics.

Table S20. Comprehensive summary of all hypothesis tests.

| **Null hypothesis (H₀)** | **Test statistic** | **df** | **p-value** | **Decision** |
| --- | --- | --- | --- | --- |
| Cost distribution is Gaussian | Park λ = 3.50 | 1 | <0.001 | H₀ rejected (Gamma family selected) |
| Gender does not affect daily cost | Wald z = 3.79 | 1 | <0.001 | H₀ rejected |
| Age does not affect daily cost | Wald z = −4.68 | 1 | <0.001 | H₀ rejected |
| Severity does not affect daily cost | Wald χ² = 0.49 | 2 | 0.78 | H₀ not rejected |
| Transplant type does not affect daily cost | Wald χ² = 1.30 | 2 | 0.52 | H₀ not rejected |
| Organ involvement does not affect daily cost | Wald z = −0.16 | 1 | 0.873 | H₀ not rejected |
| Multicollinearity present (GVIF > 2) | Max GVIF^(1/2·df)^ = 1.11 | — | — | H₀ not rejected (no collinearity) |
| Results unstable under trimming | Cook's D | — | — | H₀ not rejected (results stable) |
| Severity does not affect mortality | Log-rank χ² = 1.4 | 2 | 0.5 | H₀ not rejected |

Individual predictor tests use HC1 robust standard errors from the primary time-adjusted model (N = 50). Joint tests for severity and transplant type use Wald χ² statistics computed from the robust (HC1) covariance matrix. Multicollinearity assessed via Generalized VIF (threshold GVIF^(1/2·df) = 2.0). Stability assessed using Cook's D trimming with a < 20% CR-change criterion.

The hypothesis-testing summary shows a consistent pattern. Two predictor effects were statistically significant in the time-adjusted model. Male sex increased daily cost by 116%, and each additional year of age decreased daily cost by 3.0%. Disease severity (joint Wald p = 0.78), transplant type (p = 0.52), and organ involvement (p = 0.873) were not associated with daily cost intensity, indicating that the higher cumulative cost of more severe disease operates through a longer treatment course rather than a higher cost per day. Mortality did not differ significantly across severity groups. The absence of multicollinearity and the stability of the estimates under influential-observation removal support the robustness of the primary findings (Table S20).

Section 8: Statistical Software and Reproducibility

All statistical analyses were performed using R (version 4.5.2) running on a 64-bit Windows operating system. Table S21 lists the primary R packages employed, along with their versions and specific analytical purposes.

Table S21. R packages used for statistical analysis.

| **Package** | **Version** | **Purpose** |
| --- | --- | --- |
| stats (base R) | — | GLM estimation (Gamma family with log link) |
| sandwich | 3.1-1 | HC1 heteroscedasticity-consistent robust standard errors |
| lmtest | 0.9-40 | Coefficient testing with robust covariance matrices |
| car | 3.1-3 | Generalized Variance Inflation Factors (vif function) |
| aod | 1.3.3 | Wald χ² tests for joint significance of categorical predictors |
| MASS | 7.3-61 | Auxiliary statistical functions |
| moments | 0.14.1 | Skewness and kurtosis calculation |
| haven | 2.5.5 | Importing SPSS (.sav) data files |
| ggplot2 | 3.5.1 | Publication-quality statistical graphics |
| patchwork | 1.3.0 | Multi-panel figure composition |
| cowplot | 1.2.0 | Legend extraction and plot alignment |
| dplyr | 1.1-4 | Data manipulation and transformation |
| tidyr | 1.3.2 | Data reshaping (wide-to-long conversion) |
| scales | 1.4.0 | Axis label formatting (dollar and comma formats) |
| readxl | 1.5.0 | Importing Excel (.xlsx) data files |
| survival | 3.8-6 | Kaplan–Meier estimation and Cox proportional-hazards models |

R Core Team (2025). R: A language and environment for statistical computing. R Foundation for Statistical Computing, Vienna, Austria. https://www.R-project.org/

The analytical code supporting the findings of this study is available from the corresponding author upon reasonable request (Table S21).

Section 9: Mortality and Follow-up Analyses

This section documents the follow-up experience of the cohort and the exploratory analyses relating cGVHD severity to mortality. The study was designed to characterize the determinants of direct medical cost rather than to establish survival differences, so the survival analyses reported here are exploratory, and the small number of observed deaths limits the precision of the estimates. Follow-up duration also defines the observation window that underlies the log-time offset in the primary cost model, and its distribution is summarized in Table S23.

Overall, 11 of 50 patients (22.0%) died during follow-up. Mortality increased across severity strata, from 0 of 6 patients with Score 1 disease to 2 of 13 (15.4%) with Score 2 and 9 of 31 (29.0%) with Score 3, although the difference between strata did not reach statistical significance.

Table S22. Mortality by cGVHD severity.

| **cGVHD severity** | **Patients (n)** | **Deaths (n)** | **Mortality (%)** |
| --- | --- | --- | --- |
| Score 1 (mild) | 6 | 0 | 0.0 |
| Score 2 (moderate) | 13 | 2 | 15.4 |
| Score 3 (severe) | 31 | 9 | 29.0 |
| Overall | 50 | 11 | 22.0 |

*Mortality was assessed over the full follow-up period, and percentages are within-severity mortality. N = 50 with 11 deaths.*

The Kaplan–Meier survival curves stratified by severity are shown in Figure S5, and the log-rank test did not indicate a significant difference in survival across severity groups (χ² = 1.4, df = 2, p = 0.500). An exploratory Cox proportional-hazards model, taking severe disease (Score 3) as the reference, estimated a hazard ratio of 0.557 for moderate disease (Score 2) relative to severe disease (95% CI 0.116 to 2.674, p = 0.465) and a hazard ratio of 0.997 per year of age (p = 0.889), with a concordance of 0.635 and a likelihood-ratio test p = 0.5. An exploratory logistic regression of mortality produced an odds ratio of 0.440 for Score 2 relative to Score 3 (95% CI 0.080 to 2.436) and an odds ratio of 1.002 per year of age (95% CI 0.952 to 1.055). Patients with Score 1 disease were omitted from both multivariable models because the absence of deaths in this stratum produced complete separation. These survival findings are exploratory and should be interpreted with caution given the limited number of events.

Figure S5. Kaplan–Meier survival curves by cGVHD severity.

The observation window for each patient was defined as the interval between the initiation of cGVHD management and the last recorded follow-up assessment, and its logarithm served as the offset in the primary cost model. Follow-up was extensive and varied considerably across the cohort, as summarized in Table S23.

Table S23. Follow-up and observation-window summary.

| **Statistic** | **Value** |
| --- | --- |
| Median follow-up | 724 days |
| Interquartile range | 384–1206 days |
| Minimum | 66 days |
| Maximum | 2037 days |

*Observation window measured in days from the initiation of cGVHD management to the last follow-up assessment. N = 50.*

References for Supplementary Material

Cribari-Neto F (2004) Asymptotic inference under heteroskedasticity of unknown form. Computational Statistics & Data Analysis 45(2):215–233. <https://doi.org/10.1016/S0167-9473(02)00366-3>

Deb P, Norton EC (2018) Modeling health care expenditures and use. Annual Review of Public Health 39:489–505. <https://doi.org/10.1146/annurev-publhealth-040617-013517>

Fox J, Weisberg S (2019) An R Companion to Applied Regression, Third Edition. Sage, Thousand Oaks CA. <https://www.john-fox.ca/Companion/>

Komsta L, Novomestky F (2022) moments: Moments, Cumulants, Skewness, Kurtosis and Related Tests. R package version 0.14.1. <https://doi.org/10.32614/CRAN.package.moments>

Lesnoff M, Lancelot R (2012) aod: Analysis of Overdispersed Data. R package version 1.3.3. <https://cran.r-project.org/package=aod>

MacKinnon JG, White H (1985) Some heteroskedasticity-consistent covariance matrix estimators with improved finite sample properties. Journal of Econometrics 29(3):305–325. <https://doi.org/10.1016/0304-4076(85)90158-7>

Manning WG, Mullahy J (2001) Estimating log models: to transform or not to transform? Journal of Health Economics 20(4):461–494. <https://doi.org/10.1016/S0167-6296(01)00086-8>

Park RE (1966) Estimation with heteroscedastic error terms. Econometrica 34(4):888. <https://doi.org/10.2307/1910108>

Pedersen TL (2025) patchwork: The Composer of Plots. R package version 1.3.2. <https://doi.org/10.32614/CRAN.package.patchwork>

R Core Team (2025) R: A language and environment for statistical computing. R Foundation for Statistical Computing, Vienna, Austria. <https://www.R-project.org/>

Venables WN, Ripley BD (2002) Modern Applied Statistics with S, Fourth Edition. Springer, New York. <https://doi.org/10.1007/978-0-387-21706-2>

Wickham H (2016) ggplot2: Elegant Graphics for Data Analysis. Springer-Verlag, New York. ISBN 978-3-319-24277-4. <https://ggplot2.tidyverse.org>

Wickham H, Francois R, Henry L, Muller K, Vaughan D (2023) dplyr: A Grammar of Data Manipulation. R package version 1.1.4. <https://doi.org/10.32614/CRAN.package.dplyr>

Wickham H, Miller E, Smith D (2025) haven: Import and Export 'SPSS', 'Stata' and 'SAS' Files. R package version 2.5.5. <https://doi.org/10.32614/CRAN.package.haven>

Wickham H, Pedersen T, Seidel D (2025) scales: Scale Functions for Visualization. R package version 1.4.0. <https://doi.org/10.32614/CRAN.package.scales>

Wickham H, Vaughan D, Girlich M (2025) tidyr: Tidy Messy Data. R package version 1.3.2. <https://doi.org/10.32614/CRAN.package.tidyr>

Wilke CO (2025) cowplot: Streamlined Plot Theme and Plot Annotations for 'ggplot2'. R package version 1.2.0. <https://doi.org/10.32614/CRAN.package.cowplot>

Zeileis A (2004) Econometric computing with HC and HAC covariance matrix estimators. Journal of Statistical Software 11(10):1–17. <https://doi.org/10.18637/jss.v011.i10>

Zeileis A (2006) Object-oriented computation of sandwich estimators. Journal of Statistical Software 16(9):1–16. <https://doi.org/10.18637/jss.v016.i09>

Zeileis A, Hothorn T (2002) Diagnostic checking in regression relationships. R News 2(3):7–10. <https://CRAN.R-project.org/doc/Rnews/>

Zeileis A, Köll S, Graham N (2020) Various versatile variances: An object-oriented implementation of clustered covariances in R. Journal of Statistical Software 95(1):1–36. <https://doi.org/10.18637/jss.v095.i01>
